# Host Phenology and Geography as Drivers of Differentiation in Generalist Fungal Mycoparasites

**DOI:** 10.1371/journal.pone.0120703

**Published:** 2015-03-24

**Authors:** Alexandra Pintye, Jeanne Ropars, Nick Harvey, Hyeon-Dong Shin, Christel Leyronas, Philippe C. Nicot, Tatiana Giraud, Levente Kiss

**Affiliations:** 1 Plant Protection Institute, Centre for Agricultural Research, Hungarian Academy of Sciences (MTA), Budapest, Hungary; 2 CNRS (Centre National de la Recherche Scientifique), Ecologie, Systematique et Evolution (ESE), Orsay, France; 3 Univ Paris Sud, Ecology, Systematique et Evolution (ESE), Orsay, France; 4 Genetic Marker Services, 7 Brighton, United Kingdom; 5 Division of Environmental Science and Ecological Engineering, College of Life Sciences and Biotechnology, Korea University, Seoul, Republic of Korea; 6 Institut National de la Recherche Agronomique (INRA), Unite de Recherche UR407, Unité de Pathologie Végétale, Domaine St. Maurice, Montfavet, France; Nanjing Agricultural University, CHINA

## Abstract

The question as to why parasites remain generalist or become specialist is a key unresolved question in evolutionary biology. *Ampelomyces* spp., intracellular mycoparasites of powdery mildew fungi, which are themselves plant pathogens, are a useful model for studies of this issue. *Ampelomyces* is used for the biological control of mildew. Differences in mycohost phenology promote temporal isolation between sympatric *Ampelomyces* mycoparasites. Apple powdery mildew (APM) causes spring epidemics, whereas other powdery mildew species on plants other than apple cause epidemics later in the season. This has resulted in genetic differentiation between APM and non-APM strains. It is unclear whether there is genetic differentiation between non-APM *Ampelomyces* lineages due to their specialization on different mycohosts. We used microsatellites to address this question and found no significant differentiation between non-APM *Ampelomyces* strains from different mycohosts or host plants, but strong differentiation between APM and non-APM strains. A geographical structure was revealed in both groups, with differences between European countries, demonstrating restricted dispersal at the continent scale and a high resolution for our markers. We found footprints of recombination in both groups, possibly more frequent in the APM cluster. Overall, *Ampelomyces* thus appears to be one of the rare genuine generalist pathogenic fungi able to parasitize multiple hosts in natural populations. It is therefore an excellent model for studying the evolution of pathogens towards a generalist rather than host-specific strategy, particularly in light of the tritrophic interaction between *Ampelomyces* mycoparasites, their powdery mildew fungal hosts and the mildew host plants.

## Introduction

The question of why parasites remain generalist or become specialist remains a key unresolved issue in evolutionary biology. Generalist parasites have access to a larger number of individual hosts, but there may be trade-offs impeding optimal adaptation to several host species at the same time [1–3]. These trade-offs may take the form of constraints or a lower speed of coevolution when tracking a particular host species [4]. Specialist species may therefore be at an advantage, as they should exploit their hosts more efficiently, but this is likely to be the case only if the principal host is sufficiently abundant [5]. For specialization to occur, there must also be a mechanism preventing gene flow between sympatric parasite species adapting to different hosts, to avoid the break-up of adaptive combinations of alleles [6–8]. If this condition is not fulfilled, specialization cannot evolve and parasites remain generalists on multiple sympatric hosts. The mechanisms underlying reproductive isolation in parasites include mate choice and intersterility [9], as in free-living organisms, but also temporal isolation due to differences in host phenology [10, 11], mating within hosts after growth [12–15] and selfing [8, 16].

It is therefore not straightforward to predict whether and when a parasite will remain generalist or evolve towards specialization. Many case studies are required before we can draw general inferences. True generalist parasites appear to be rare; morphological species long considered generalist are often found to be cryptic specialist species in studies based on molecular markers [8, 17, 18]. However, genuine generalist parasites do exist, and this group includes the fungi *Botrytis cinerea*, *Sclerotinia sclerotiorum*, and *Batrachochytrium dendrobatidis* [19–22].

Geography is another driver of genetic differentiation in parasites. Spatial genetic structure has been detected in a number of parasites, even in fungi with wind-borne spores [15, 23–29]. Spatial genetic structure can be used to infer dispersal distances, which is useful for the monitoring and prediction of epidemics caused by pathogenic fungi [23, 24, 30–32]. In this respect, however, our current knowledge on fungal pathogens is relatively limited, especially in the case of mycoparasites, *i*.*e*., fungi parasitizing other fungi, with only a few studies available, on tremellalean mycoparasites [33] and some lichenicolous fungi [34, 35].

*Ampelomyces* is an interesting biological model in this concept. These fungi are intracellular mycoparasites of powdery mildew fungi, which are themselves plant pathogens [36, 37]. Differences in host phenology promote temporal isolation between sympatric mycoparasites infecting apple powdery mildew (*Podosphaera leucotricha*) and those infecting other powdery mildew species on host plants other than apple [11]. Indeed, apple powdery mildew (APM) epidemics occur in spring, whereas the fungal hosts of the other *Ampelomyces* species cause epidemics mostly in the summer and fall. Consequently, *Ampelomyces* populations infecting APM display strong differentiation from mycoparasites infecting other mildews, despite their ability to infect these other mildews in laboratory and field experiments [11]. However, it was not possible to investigate genetic differentiation between the *Ampelomyces* parasites of different mildews species causing epidemics in summer and fall in these previous studies. Indeed, the microsatellite markers developed for the mycoparasites of apple powdery mildew gave no amplification in *Ampelomyces* strains isolated later in the season, because the level of genetic differentiation was too high. Gene sequences revealed no clear separation according to host species in non-APM *Ampelomyces* [38–41] but differentiation may have occurred too recently for detection by this approach.

No strong geographic structure was found in our previous study focusing on European *Ampelomyces* strains from apple powdery mildew [11]. Microsatellite markers revealed long-distance spread across Europe, consistent with the wind dispersal of these mycoparasites within airborne parasitized powdery mildew spores (Szentivanyi & Kiss 2003; Kiss 2008). However, further knowledge of the geographical structure and dispersal ability of *Ampelomyces* populations is required, especially because *Ampelomyces* strains are sold as biological control agents [42].

Another interesting question in *Ampelomyces* is the extent of recombination. Sexual structures have never been observed in the field, but some footprints of recombination have been detected in *Ampelomyces* from apple powdery mildew [11]. Recombination traces have proved to be common in fungal species previously thought to be asexual [43–51], reflecting rare sexual events or the occurrence of sex in places or at times not accessible to observation. We were unable to investigate the occurrence of sex in *Ampelomyces* from hosts other than apple powdery mildew, because of the lack of polymorphic markers [11].

We report here the development of new microsatellite markers for mycoparasites isolated from powdery mildew fungi in the summer and fall, and their use to genotype a large collection of mycoparasites from a number of powdery mildew species and host plants, worldwide but with a particular effort in Europe. We addressed the following questions: 1) Is there any genetic differentiation between *Ampelomyces* strains isolated from different host plants affected by mildew epidemics in the summer and fall that might indicate specialization? 2) Is there any geographic structure within *Ampelomyces*? 3) Are there footprints of recombination in *Ampelomyces*, within and between powdery mildew hosts?

## Materials and Methods

### Fungal materials

We studied 469 *Ampelomyces* strains maintained in culture and 168 dried powdery mildew-infected leaf samples containing *Ampelomyces* pycnidia (S1 Table). Leaf samples were obtained as described by Kiss *et al*. (2011) and each was treated as a single *Ampelomyces* strain (haplotype) on the basis of microsatellite genotyping results [52]. We thus studied 637 *Ampelomyces* strains in total. Twelve of these strains were newly obtained from *Blumeria graminis*, the only powdery mildew species known to infect various monocot species. These new strains were isolated as described previously [40]. The rest of the strains came from previous works [11, 38, 40, 41, 53–55] (S1 Table). Most of these strains, 157 in total, came from *Arthrocladiella mougeotii*, a powdery mildew species infecting a solanaceous weed, *Lycium halimifolium*. A group of 17 strains were isolated from *Erysiphe necator* (formerly *Uncinula necator*) infecting grapevine. The remaining strains came from powdery mildew species infecting various host plant species, none of which was widely sampled. Overall, our dataset of 637 *Ampelomyces* strains contained 394 strains infecting apple powdery mildew (APM) and 243 non-APM strains. We chose *B*. *graminis*, *A*. *mougeotii* and *E*. *necator* to be the most intensively sampled non-APM mycohost species because these three powdery mildews have been shown to be representative of the diversity of non-APM species [56].

### DNA extraction

Total genomic DNA was extracted from about 10 to 15 mg of freeze-dried mycelium for each *Ampelomyces* strain and from about 5 to 10 mg of dried leaf sample for samples containing *Ampelomyces* pycnidia. We used the DNeasy Plant Mini Kit (Qiagen) for DNA extraction.

### Development of microsatellite markers

Microsatellite markers were identified as described previously [52], using 11 strains (highlighted in S1 Table) isolated in the summer or fall, from different mycohosts in Europe, Israel and China. They belonged to different ITS and actin gene clades [38]. The genotypes of all the strains tested in this work are given in S2 and S3 Tables.

### Population genetic analyses

**Microsatellite genotyping.** Amplifications were carried out with forward primers labeled with fluorescent FAM or HEX dyes (Sigma Aldrich). Each PCR was performed in a final volume of 10 μl containing 5 μl Multiplex PCR Master Mix (Qiagen) and 0.2 μl of each primer at a concentration of 10 μM and 2 μl of genomic DNA. The primer sequences are shown in Table 1. Three primer combinations were used: set 1 (LK3c and LK7d), set 2 (LK3a, LK7c and LK10c) and set 3 (LK3b, LK7f and LK10d). Assays were carried out in the following conditions: initial denaturation for 5 minutes at 95°C, followed by 28 cycles of denaturation for 45 s at 95°C, annealing for 50 s at 54°C, extension for 1 minute at 72°C, and a final extension for 5 minutes at 72°C. PCR products were heated at 94°C for 5 minutes, chilled on ice and separated on an ABI Prism 3130XL Genetic Analyzer (Applied Biosystems). Each gel contained GenScan-500LIZ Size Standards (35 to 500 bp, Applied Biosystems), for the analysis of microsatellite data with GeneMapper Software (Applied Biosystems).

**Table 1:** Characteristics of the eight polymorphic microsatellite loci identified in *Ampelomyces* strains isolated from many different mycohosts in autumn.

Locus	Motif	Primer sequence (5'-> 3')	Allele size range	No. ofalleles
LK3a	[CAGCAGCA-_n_15]_8_	CAAGATCTGCCGCCAACC	203–302	25
		GGTGTTGTTGTGCATGTTGTC		
LK3b	[cgg]_5_	CGGCACGAAATCTACCTGTC	126–144	5
		CGCAGAGGTGGATGTAGGTT		
LK7c	[ggt/gga/ggc]_3_	GATAGGACGCGGTTATGGAA	176–221	9
		GATGTGGCTTCCTGGTTCG		
LK10c	[gct]_6_[cgg]_3_	CATCGTGATGTTCTGGGTGA	149–197	10
		AGACCACAATCTCCGACCAG		
LK10d	[ggt]_5_[ggcggt]_3_	CAGAAGTGGATTGCGGAGAG	179–257	21
		CAAGGCCACATCCAAGTTCT		
LK3c	[aag]_13_	GACAAGAAACCTTGGGTTGG	101–161	20
		GGACGACGATTTGCAGACTA		
LK7d	[caccgc]_4_	GCTTCGGGTTTGTCTCAGTC	146–188	7
		CGAAAGGGTTGATGAGGTTT		
LK7f	[gt]_12_	AAAAGTCAAGGGACCACACG	181–253	23
		CATAAGCGATGGGAGTTGGT		

The first five loci listed below were also used to genotype *Ampelomyces* strains isolated from apple powdery mildew (APM) in spring.

**Genetic analyses: SplitsTree, correspondence analyses, linkage disequilibrium, and structure.** SplitsTree 4 [57] (http://www.splitstree.org/) was used to visualize differentiation and the occurrence of recombination, (i) for the total dataset, with the five microsatellite markers yielding amplification products for all samples, and (ii) on the 209 non-APM strains that could be genotyped with all eight markers. Factorial correspondence analyses (FCA) were performed with GENETIX v4.05 [58]. F_ST_ and linkage disequilibrium between the five markers and population differentiation were assessed online with Genepop [59, 60], http://genepop.curtin.edu.au/. We used the Genclone software [61] to determine the number of different multilocus genotypes (MLGs) in the datasets and for the estimation, by permutation, of the expected number of MLGs for different numbers of markers.

We used the individual-based Bayesian clustering methods implemented in STRUCTURE 2.3.3 [62] to infer population structure. STRUCTURE makes use of Markov Chain-Monte Carlo (MCMC) simulations to infer the proportion of ancestry of genotypes from *K* distinct clusters. The underlying algorithms attempt to minimize deviations from Hardy–Weinberg and linkage disequilibria. Ten independent analyses were carried out for each number of clusters, from *K* = 2 to *K* = 10, with admixture models and 500,000 MCMC iterations, after a burn-in of 500,000 steps. Outputs were processed with CLUMPP v1.1.2 [63], to identify clustering solutions in replicated runs for each value of *K*. Population structure was then displayed graphically with DISTRUCT v1.1 [64]. We used the Evanno method, via the STRUCTURE Harvester website (http://taylor0.biology.ucla.edu/structureHarvester/) [65], to identify the *K* value corresponding to the strongest structure.

## Results

### Development and characteristics of new microsatellite markers

We identified and developed eight new microsatellite markers (Table 1) using 11 *Ampelomyces* strains isolated from different mycohosts during the fall (S1 Table). Five of these eight new markers, LK7c, LK3a, LK10c, LK3b and LK10d, amplified the target loci in all the APM and non-APM strains studied (S2 Table), regardless of the season in which they were isolated. The number of alleles was found smaller in APM strains than in non-APM strains (Table 2). The remaining three markers could be amplified from only 209 strains, all of which were non-APM strains belonging to different lineages according to their ITS and actin gene sequences [38–40]. Most of these phylogenetically diverse strains were isolated in summer or fall, in different parts of the world (China, France, Germany, Hungary, Israel, Italy, Korea, South Africa, the United Kingdom and the USA); the season in which isolation occurred was not known for a few strains (S1 Table).

**Table 2:** Number of alleles detected at the five microsatellite markers in 394 *Ampelomyces* strains isolated in spring from apple powdery mildew (APM strains), and in 243 non-APM strains isolated later in the season from many other powdery mildew species infecting various host plants.

Strain type			Locus		
	LK7c	LK3a	LK10c	LK3b	LK10d
APM strains	1	5	4	2	3
non-APM strains	8	20	7	3	18

The microsatellite profiles of the strains are shown in S2 Table.

### Genetic diversity and population structure in *Ampelomyces* strains

**Fig 1:**
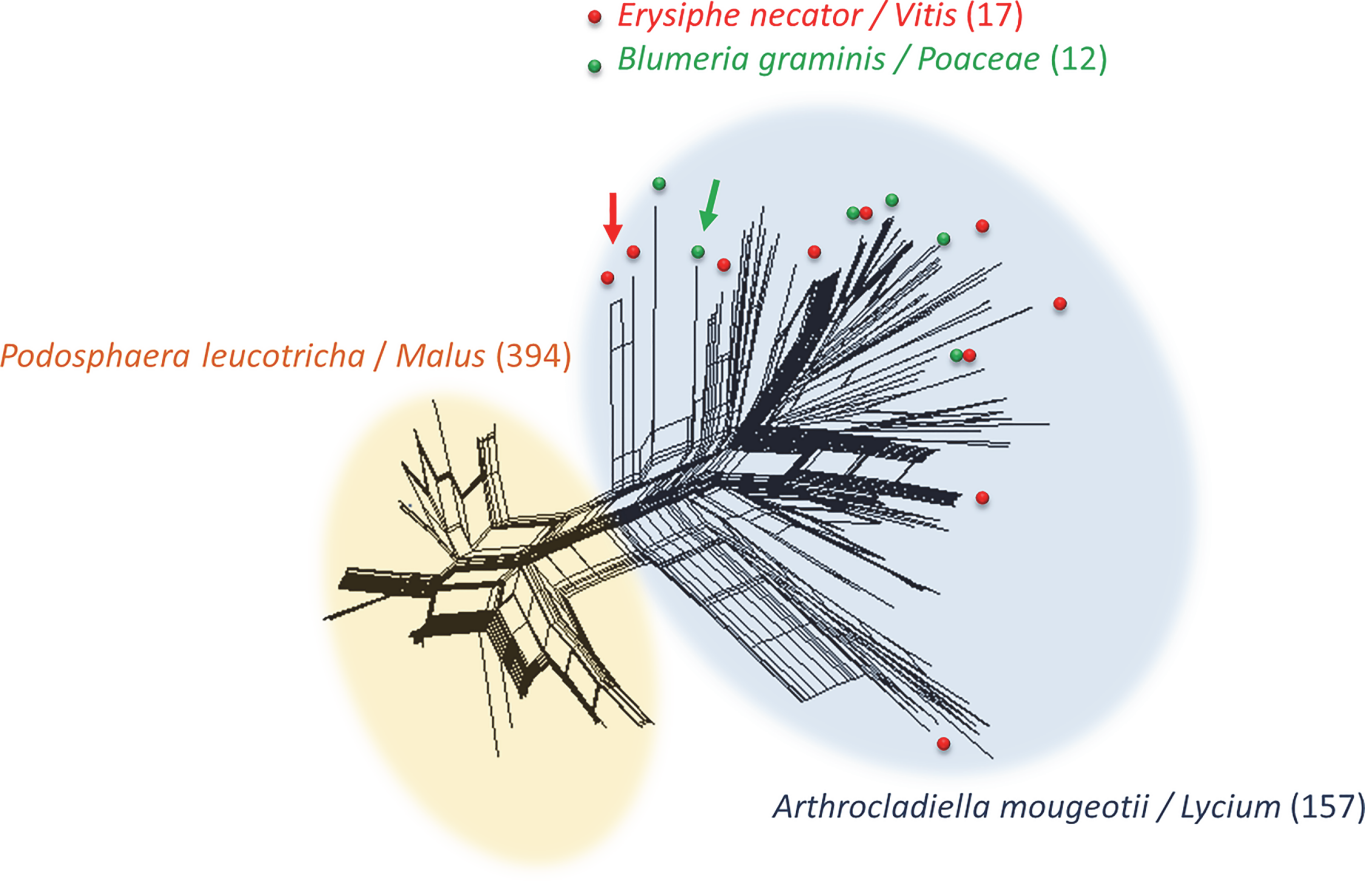
SplitsTree analysis of all 637 *Ampelomyces* strains, based on five microsatellite markers. Widely sampled fungal hosts and the plant hosts from which they were collected are indicated by colors and the number of strains is indicated in brackets. Two main clusters were identified, one consisting of the 394 strains from apple powdery mildew (APM, *Podosphaera leucotricha*, light orange cloud), isolated in spring, and the other comprising all the non-APM strains, *i*.*e*. the 157 strains isolated from *Arthrocladiella mougeotii* infecting *Lycium halimifolium* plants in Hungary (light blue cloud) and the strains isolated from other powdery mildew hosts in Europe and elsewhere, later in the season. In this second cluster, positions are shown by arrows for the 17 strains isolated from grapevine powdery mildew (*Erysiphe necator*, in red) and the 12 strains isolated from *Blumeria graminis* infecting wild grass species (in green). Reticulation indicates the occurrence of recombination.

**Fig 2:**
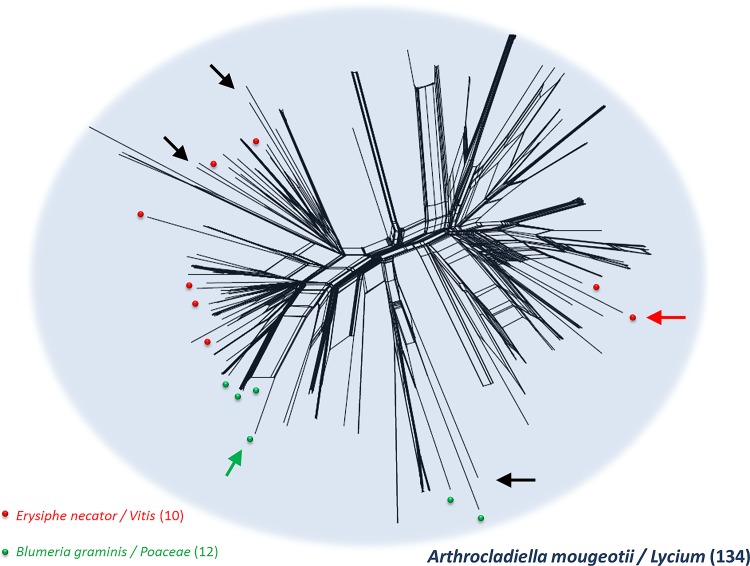
SplitsTree analysis of the 209 non-APM *Ampelomyces* strains in which the eight microsatellite markers could be amplified. Widely sampled fungal hosts and the plant hosts from which they were collected are indicated by colors and the number of strains is shown in brackets: 134 strains came from *Arthrocladiella mougeotii* infecting *Lycium halimifolium* plants (blue cloud), 12 strains came from *Blumeria graminis* infecting grasses (green points), 10 strains from grapevine powdery mildew (*Erysiphe necator*) (red points), and 53 other strains were isolated from several other powdery mildew species infecting various host plants, isolated in summer and fall. The position of an *Ampelomyces* strain isolated from *E*. *necator* in the USA and that of another strain isolated from *B*. *graminis* in Korea are indicated by red and green arrows, respectively. Black arrows indicate the positions of the other three non-European strains. Reticulation indicates the occurrence of recombination.

**Fig 3:**
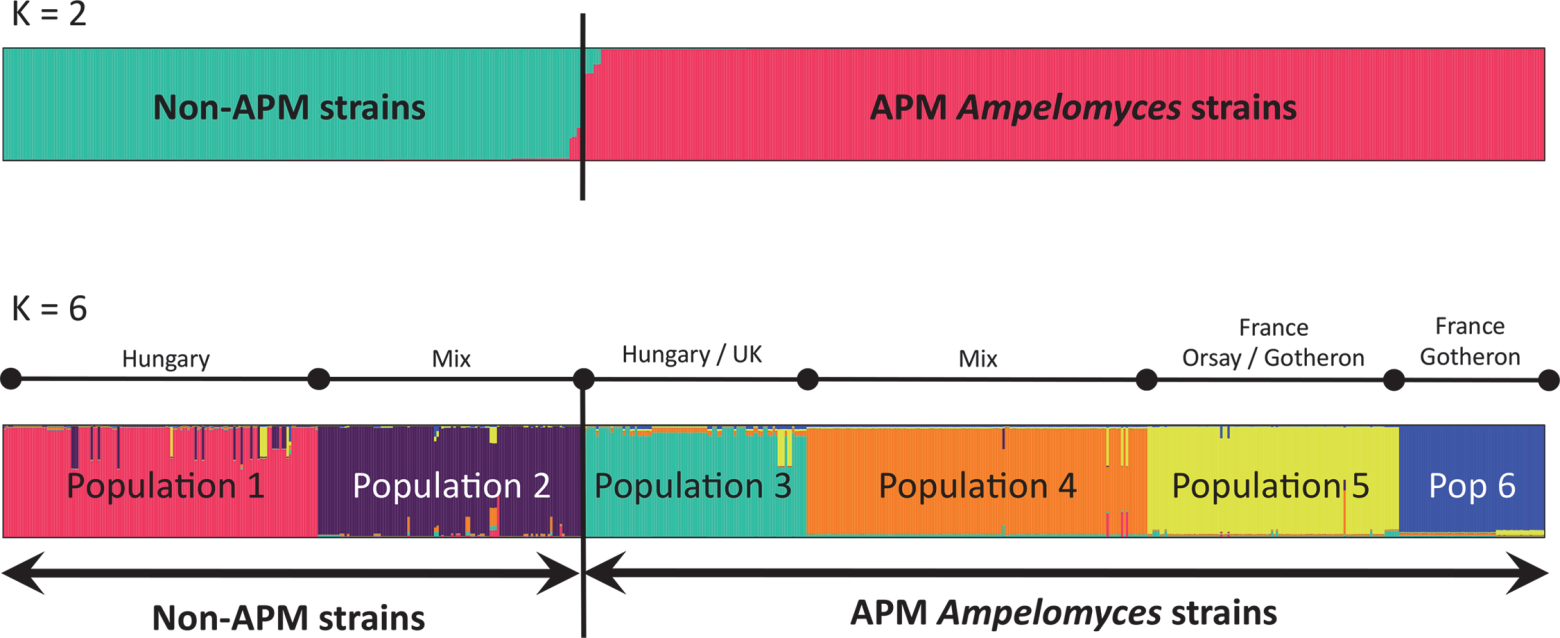
Population structure in *Ampelomyces*. Structure was inferred by STRUCTURE for *K* = 2 and *K* = 6 (see S1 Fig. for the bar plots corresponding to other *K* values). STRUCTURE yielded well defined clusters up to *K* = 6, indicating the existence of six genetically differentiated clusters. *K* = 2 separates APM and non-APM strains. The geographical origin of strains is indicated on the *K* = 6 barplots.

We first investigated the population structure of *Ampelomyces* strains with STRUCTURE [66]. SplitsTree analyses were also carried out, separately for the genotypes of all the strains based on five microsatellite markers (Fig. 1), and for the 209 non-APM strains genotyped with the eight markers (Fig. 2). STRUCTURE yielded well defined clusters at *K* values of up to 6 (Fig. 3 and S1 Fig.), indicating the existence of six genetically differentiated clusters. For values of *K* of 7 and above, each new cluster included only admixed genotypes, indicating a lack of further structure. The deltaK value [67] confirmed that the split into six populations corresponded to a peak in the strength of the structure in the dataset, the strongest peak being at *K* = 4 (S2 Fig.). At *K* = 2, apple powdery mildew (APM) strains were separated from all other (non-APM) strains. This differentiation between APM and non-APM strains also appeared clearly on the SplitsTree (Fig. 1). At *K* = 6, non-APM strains were split into two clusters (Fig. 3, populations 1 and 2), whereas strains isolated from apple powdery mildew were split into four clusters (Fig. 3, populations 3 to 6). The clusters of non-APM strains did not correspond to either different host mildews or different host plants, but some geographical clustering was revealed: one of the clusters encompassed most of the Hungarian strains (128 of 130), together with two Italian strains, whereas the second cluster contained strains of more diverse origins, from Hungary, France, the United Kingdom, China, the USA, and Israel. Some geographical structure was also observed within APM strains: populations 5 and 6 mostly included strains from France, together with 13 of the 104 strains from Hungary (in population 5), and 3 of the 60 strains from the UK (in the population 6), whereas populations 3 and 4 included strains from different parts of Europe (Hungary, France, Germany, and the UK). On the other hand, the non-European strains appeared scattered in the SplitsTree (arrows on Fig. 2).

The existence of six genetically divergent populations was confirmed by F_ST_ analyses. The fixation index was 0.25 between the non-APM populations (populations 1 and 2), indicating strong genetic differentiation. Pairwise fixation indices ranged between 0.22 and 0.42 between the other four populations, indicating that there was also strong genetic differentiation between APM populations. The level of genetic differentiation was strongest between APM and non-APM clusters, with an F_ST_ value of 0.49 between the two clusters obtained at *K* = 2.

A factorial correspondence analysis (FCA) yielded further support for the genetic patterns revealed above (S3 Fig.), with 7.82%, and 4.38% of the variance explained by axes 1 and 4, respectively). APM strains and non-APM strains appeared to be strongly separated along axis 1. The other populations were also segregated, but less strongly.

### Recombination footprints

In the dataset based on five markers and 637 strains, only 93 MLGs were detected, with a mean of 6.7 strains per MLG. These identical MLGs probably did not correspond entirely to clones, resulting instead from the insufficiently high discriminatory power of the five markers, as a permutation estimating the number of MLGs as a function of the number of markers used did not reach a plateau (S4 Fig.); actually, most of these identical MLGs could be differentiated using eight markers. In the dataset based on eight markers and 209 strains, 91 MLGs were detected, with a mean of only 2.3 strains per MLG, and a single abundant MLG for 17 strains in the APM cluster. In this analysis, the number of MLGs as a function of the number of markers used did begin to reach a plateau (S4 Fig.), although the shape of the curve indicated that discrimination could probably be increased further by the use of a larger number of markers. Thus, the identical MLGs obtained probably do not strictly correspond to clones and did not, therefore, bias the structure analyses.

We assessed the occurrence of sex, by looking for footprints of recombination in the split decomposition analysis, in which recombination events were identified as reticulations (Figs. 1 and 2). The SplitsTree analyses yielded networks with long branches, but also with some reticulations, mostly within each of the APM and non-APM clusters, with a smaller number between APM and non-APM strains. This pattern is indicative of pervasive clonality with occasional recombination events, these events being less frequent between than within the APM and non-APM clusters, confirming that differences in host plant phenology impede gene flow between these two groups. The branches appeared to be shorter and the reticulations more frequent within the APM cluster than within the non-APM cluster (Fig. 1), confirming the lower level of diversity and suggesting less ancient clonality and/or more frequent recombination events. The SplitsTree analyses have also shown that there was no detectable genetic differentiation within non-AMP strains as a function of the powdery mildew host, as strains from different powdery mildew species were scattered (Figs. 1 and 2). Non-APM strains belonging to different phylogenetic lineages based on their ITS and actin gene sequences [38–40] did not form distinct groups in any of the SplitsTree analyses (Figs. 1 and 2).

Linkage disequilibrium analyses were consistent with the SplitsTree and STRUCTURE analyses, indicating frequent clonality with occasional recombination events, these events being more frequent in the APM cluster. Linkage disequilibrium was, indeed, significant within both AMP and non-APM clusters, at least partly reflecting a Wahlund effect due to the genetic structure. When the populations were considered separately, we also detected significant linkage disequilibrium within both the non-APM populations (populations 1 and 2), whereas no significant linkage disequilibrium was detected for the three APM populations for which linkage disequilibrium could be calculated.

## Discussion

In this study, we developed eight new polymorphic microsatellite markers for investigating the population structure and diversity of *Ampelomyces* strains isolated from many powdery mildew species infecting very different host plant species and representative of the genetic diversity in Erysiphales. Five out of eight of our newly developed microsatellite markers could be amplified in both AMP and non-AMP strains, which was not the case for previous microsatellite markers [11]. The newly developed markers will be useful for detecting and monitoring *Ampelomyces* strains released as commercial biological control agents in vineyards [42] and in agricultural fields, because they were amplified reliably from environmental samples as well as from strains maintained in culture.

Various analyses, using a comprehensive dataset with the genotypes of APM and non-APM strains, confirmed that there was a high level of genetic differentiation between these two groups, with no evidence of gene flow. This is consistent with the findings of our previous study, indicating that APM strains form a distinct cryptic species, genetically isolated from non-APM strains by temporal isolation due to differences in the phenology of their powdery mildew hosts [11]. Indeed, apple powdery mildew overwinters in apple buds [41] and begins to sporulate soon after bud burst, becoming widespread on its host plants in spring and persisting without much further spread, mostly on green shoots, during the summer and fall. By contrast, the other powdery mildew species, the mycohosts of the non-APM strains, begin to sporulate and spread on their respective host plants later in the season. They also continue to display active sporulation and spread on host plants until late fall [11]. Phylogenetic analyses have previously suggested that the non-APM strains could be classified into distinct molecular taxonomic units (MOTUs), which were not specific to particular mycohost or plant host species [38–40, 53]. Neither SplitsTree nor STRUCTURE analyses however confirmed here the distinction of the non-APM strains according to the MOTUs defined in these previous works [38–40, 53].

STRUCTURE analyses further revealed that the *Ampelomyces* strains included in this work could be split into six distinct populations (Fig. 3). The non-APM strains formed two genetically differentiated populations that could be accounted for by geography, as one of the clusters contained mostly Hungarian strains. This differentiation of Hungarian strains probably reflects their abundance in the dataset, and further geographical clustering may be revealed within the other clusters after additional sampling. The APM strains formed four different populations that also displayed some geographical clustering. Most of the strains, both APM and non-APM, came from Hungary and two regions of France, again potentially explaining why STRUCTURE differentiated these three regions. This study provides the first evidence of geographical structure in *Ampelomyces*, confirming that, even in wind-dispersed fungi “not everything is everywhere”, and that gene flow is restricted by distance, as shown in many other fungi [15, 23–29]. The ability of our markers to reveal geographical structure in European populations highlights their discriminatory power and indicates that the lack of structure according to host is real and not due to low power.

The analyses of non-APM *Ampelomyces* populations detected no structure according to powdery mildew mycohost and/or host plant species, even in the most widely sampled areas. Moreover, strains isolated from different powdery mildew species, infecting different host plants, sometimes had identical, or very similar, microsatellite profiles. These results suggest that there are no barriers restricting gene flow among *Ampelomyces* strains affecting different powdery mildew species, on different host plants, in the same environment. These findings suggest that this mycoparasite is a genuine generalist. This may appear contrary to the results of laboratory experiments showing that *Ampelomyces* strains from grapevine powdery mildew parasitize their original mycohost species more strongly than two other test powdery mildew species [55], or showing differences in the virulence of *Ampelomyces* strains against three powdery mildew species [68]. However, there may be some polymorphism in the ability of different strains to infect various host species or aggressiveness towards different host species within a given mycoparasite species. By contrast, other cross-inoculation experiments have repeatedly shown that a number of *Ampelomyces* strains isolated from different powdery mildew species were all able to parasitize test powdery mildew species in the laboratory [40, 41, 69] with similar intensities of mycoparasitism for strains isolated from conspecific and other powdery mildew species [11]. Moreover, a field experiment has clearly demonstrated that genetically differentiated *Ampelomyces* strains occurring naturally in certain powdery mildew species can easily disperse and parasitize other powdery mildew species on their respective host plants [11]. Furthermore, a broad sampling campaign revealed that grapevine powdery mildew was naturally parasitized by phylogenetically diverse *Ampelomyces* strains in the field [38].

Some of these previous studies, and the results reported in this work, indicate that *Ampelomyces* mycoparasites are not strictly specialized on the powdery mildew species in which they are found in the field, with each cluster instead naturally parasitizing a wide range of powdery mildew species. Moreover, this study provides the first evidence for recombination events in *Ampelomyces* strains isolated from different, non-APM mycohost species in the field. The sexual fruiting bodies of *Ampelomyces* have never been observed, despite intensive morphological studies on this mycoparasite [37]. The recombination footprints detected may, therefore, be the result of ancient sexual events, before a loss of sex, or recombination may result from a parasexual process in the hyphal anastomoses within the powdery mildew mycelium, the sole habitat of *Ampelomyces*. It may also be that sex events are very rare and were not observed so far.

Despite their interesting life style and their practical use as commercially available biological control agents for plant pathogenic fungi, very little is known about the population genetics of mycoparasites. This work, together with our earlier study (Kiss *et al*. 2011), provides the first insight into this domain, based on a reliable set of genetic tools tested with hundreds of strains, and could be used to promote the use of this natural tritrophic relationship between *Ampelomyces* mycoparasites, their powdery mildew fungal hosts, and the plant hosts of the mildews, as a model system for the study of such interactions. Furthermore, *Ampelomyces* mycoparasites appear to be among the rare genuine generalist pathogenic fungi, able to parasitize multiple hosts in natural populations. They therefore constitute an excellent model for studying the evolution of pathogens towards a generalist rather than a host-specific strategy [1–3].

## Supporting Information

S1 Fig Population structure in *Ampelomyces*. The structure has been inferred by STRUCTURE for K = 2 to K = 7. The STRUCTURE program could form well-defined clusters up to K = 6, indicating the existence of six genetically differentiated clusters. The two different solutions found by Structure at certain K values are shown. The strains are in the same order as in Fig. 1.
(TIF)

S2 Fig Result of the Evanno method for detecting the number of K groups for which the subsequent increase in K yield less information than the previous increase in K. (TIF)

S3 Fig Factorial Correspondence Analysis (FCA) illustrating the differentiation of the six populations of *Ampelomyces* according to axes 1 and 4. The populations are defined based on STRUCTURE assignations (Fig. 1 at K = 6).
(TIF)

S4 Fig Number of MLGs (MultiLocus Genotypes) depending on the number loci considered using permutations A) for the five markers dataset, B) for the eight markers datasets. (TIF)

S1 Table Designations of strains, their fungal host and host plant species of collection, and dates and places of collection of the *Ampelomyces* strains and powdery mildew-infected apple leaf samples bearing *Ampelomyces* mycoparasites included in this study. The 11 strains used to develop the microsatellite markers are shown in red boldface. Strains of *Ampelomyces* were designated with upper case letters and/or numbers. When more than one strain was isolated from the same site/plant, these were distinguished by lower case letters (e.g., B119-a and B119-b). If available, public culture collection designations of the strains are also shown. Lower case designations (b1-b365) were applied for apple leaf samples, preserved as herbarium materials and used in the microsatellite genotyping work. Dates and places of collections given with all known details. If several strains were used, collected from more than one site within a locality, or from more than one plant individual within one site, the site and/or the plant number was shown in the table. The identities of the host fungal and host plant species of the strains obtained from earlier works were determined by their suppliers.
(PDF)

S2 Table Genotypes of *Ampelomyces* strains isolated from different mycohosts, genotyped with five microsatellite markers. (PDF)

S3 Table Genotypes of *Ampelomyces* strains isolated from different mycohosts in autumn, genotyped with eight microsatellite markers. (PDF)
