# Supplementary figures and images for: Host Phenology and Geography as Drivers of Differentiation in Generalist Fungal Mycoparasites
Source: PLoS One. 2015 Mar 24;10(3):e0120703. doi: 10.1371/journal.pone.0120703 (PMC4372539; doi:10.1371/journal.pone.0120703)

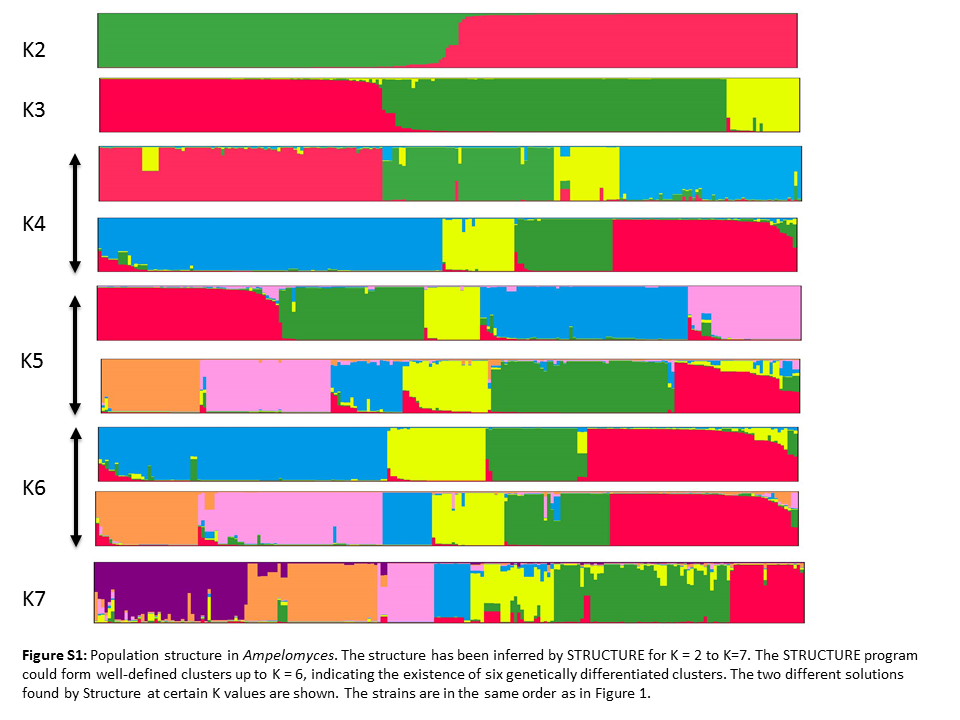

Supplement: S1 Fig — The structure has been inferred by STRUCTURE for K = 2 to K = 7. The STRUCTURE program could form well-defined clusters up to K = 6, indicating the existence of six genetically differentiated clusters. The two different solutions found by Structure at certain K values are shown. The strains are in the same order as in Fig. 1. (TIF) [file pone.0120703.s001.tif]

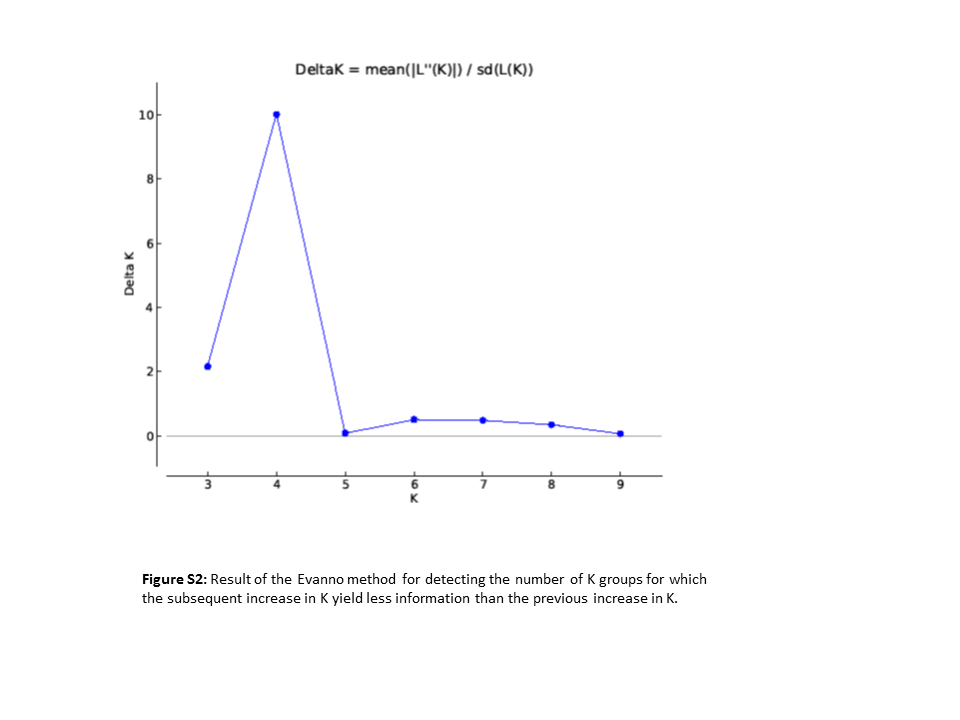

Supplement: S2 Fig — (TIF) [file pone.0120703.s002.tif]

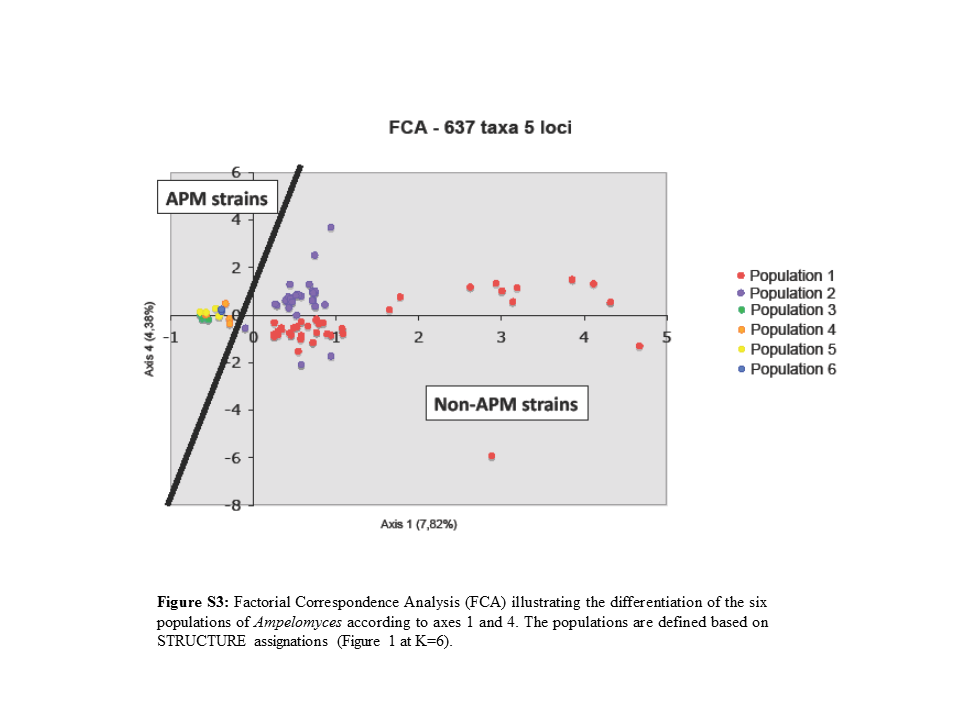

Supplement: S3 Fig — The populations are defined based on STRUCTURE assignations (Fig. 1 at K = 6). (TIF) [file pone.0120703.s003.tif]

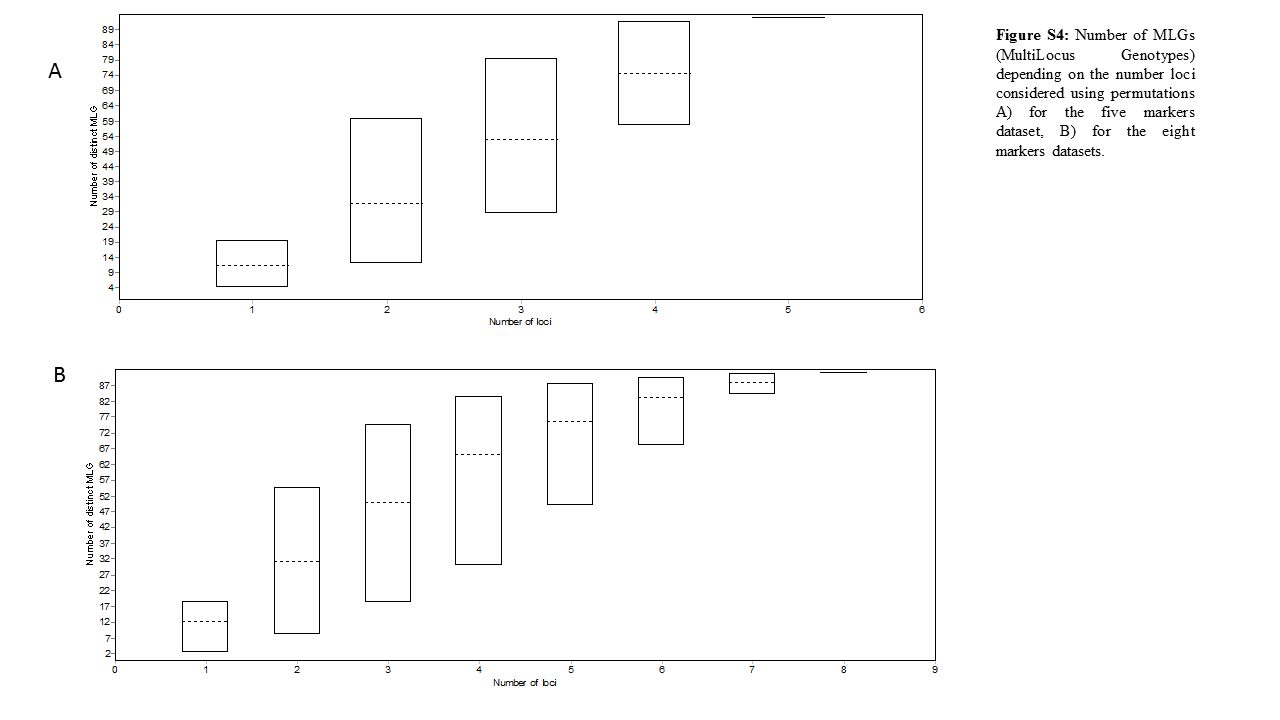

Supplement: S4 Fig — (TIF) [file pone.0120703.s004.tif]
