# Supplementary material for: Host Phenology and Geography as Drivers of Differentiation in Generalist Fungal Mycoparasites
Source: PLoS One. 2015 Mar 24;10(3):e0120703. doi: 10.1371/journal.pone.0120703 (PMC4372539; doi:10.1371/journal.pone.0120703)
Supplement: S1 Table — The 11 strains used to develop the microsatellite markers are shown in red boldface. Strains of Ampelomyces were designated with upper case letters and/or numbers. When more than one strain was isolated from the same site/plant, these were distinguished by lower case letters (e.g., B119-a and B119-b). If available, public culture collection designations of the strains are also shown. Lower case designations (b1-b365) were applied for apple leaf samples, preserved as herbarium materials and used in the microsatellite genotyping work. Dates and places of collections given with all known details. If several strains were used, collected from more than one site within a locality, or from more than one plant individual within one site, the site and/or the plant number was shown in the table. The identities of the host fungal and host plant species of the strains obtained from earlier works were determined by their suppliers. (PDF) [file pone.0120703.s005.pdf]

Dataset S1. Designations od strains, their fungal host and host plant species of collection, and dates and places of collection of the *Ampelomyces* strains and powdery mildew-infected apple leaf samples bearing *Ampelomyces* mycoparasites included in this study. The 11 strains used to develop the microsatellite markers are shown in **red boldface**. Strains of Ampelomyces were designated with upper case letters and/or numbers. When more than one strain was isolated from the same site/plant, these were distinguished by lower case letters (e.g., B119-a and B119-b). If available, public culture collection designations of the strains are also shown. Lower case designations (b1-b365) were applied for apple leaf samples, preserved as herbarium materials and used in the microsatellite genotyping work. Dates and places of collections given with all known details. If several strains were used, collected from more than one site within a locality, or from more than one plant individual within one site, the site and/or the plant number was shown in the table. The identities of the host fungal and host plant species of the strains obtained from earlier works were determined by their suppliers.

STRAIN / SAMPLE DESIGNATION (public culture collection designations in parentheses if applicable)	HOST FUNGAL SPECIES	HOST PLANT SPECIES	DATE OF COLLECTION	PLACE OF COLLECTION	GENBANK ACCESSION NUMBER OF THE nrDNA ITS SEQUENCE (reference in parentheses if determined in an earlier work)
***Ampelomyces* strains isolated from grass powdery mildew (*Blumeria graminis*)**					
BgrA	*Blumeria graminis*	unknown grass	24 August 2009	Brno, Czech Republic	
BgrB	*Blumeria graminis*	unknown grass	24 August 2009	Brno, Czech Republic	
BgrC	*Blumeria graminis*	unknown grass	24 August 2009	Brno, Czech Republic	
BgrD	*Blumeria graminis*	unknown grass	24 August 2009	Brno, Czech Republic	
BgrE	*Blumeria graminis*	unknown grass	24 August 2009	Brno, Czech Republic	
BgrF	*Blumeria graminis*	unknown grass	24 August 2009	Brno, Czech Republic	
BgrG	*Blumeria graminis*	unknown grass	24 August 2009	Brno, Czech Republic	
BgrH	*Blumeria graminis*	unknown grass	24 August 2009	Brno, Czech Republic	
BgrI	*Blumeria graminis*	unknown grass	27 August 2009	Rajec-Jestreki, Czech Republic	
KACC 43563	*Blumeria graminis*	*Diarrhena japonica*	2006	Hongcheon, Korea	
KACC 44850	*Blumeria graminis*	*Agropyron* sp.	2009	Brno, Czech Republic	
KACC 44851	*Blumeria graminis*	*Piptatherum virescens*	2009	Brno, Czech Republic	
***Ampelomyces* strains isolated from grapevine powdery mildew (*Erysiphe necator*)**					
Vitis42	*Erysiphe necator*	*Vitis vinifera*	14 Oct. 2009	Paterno, Italy	JN417725 (Pintye et al. 2012)
Vitis55	*Erysiphe necator*	*Vitis vinifera*	15 Oct. 2009	Santo Stefano, Italy	JN417732 (Pintye et al. 2012)
Vitis56	*Erysiphe necator*	*Vitis vinifera*	17 Oct. 2009	Santo Stefano, Italy	JN417733 (Pintye et al. 2012)
Vitis60	*Erysiphe necator*	*Vitis vinifera*	17 Oct. 2009	Santo Stefano, Italy	JN417734 (Pintye et al. 2012)
Vitis70 (CBS 132220)	*Erysiphe necator*	*Vitis vinifera*	16 Oct. 2009	Jesi, Italy	JN417739 (Pintye et al. 2012)
Vitis71	*Erysiphe necator*	*Vitis vinifera*	16 Oct. 2009	Jesi, Italy	
Vitis72	*Erysiphe necator*	*Vitis vinifera*	16 Oct. 2009	Portonovo, Italy	JN417740 (Pintye et al. 2012)
Vitis76	*Erysiphe necator*	*Vitis vinifera*	16 Oct. 2009	Portonovo, Italy	JN417742 (Pintye et al. 2012)
Vitis79	*Erysiphe necator*	*Vitis vinifera*	16 Oct. 2009	Portonovo, Italy	JN417743 (Pintye et al. 2012)

Vitis81	*Erysiphe necator*	*Vitis vinifera*	22 Oct. 2009	Szekszárd, Hungary	JN417744 (Pintye et al. 2012)
Vitis98	*Erysiphe necator*	*Vitis vinifera*	28 Sept. 2009	Budapest, Hungary	JN417748 (Pintye et al. 2012)
Vitis107	*Erysiphe necator*	*Vitis vinifera*	22 Sept. 2009	Eger, Hungary	JN417752 (Pintye et al. 2012)
Vitis113	*Erysiphe necator*	*Vitis vinifera*	22 Sept. 2009	Eger, Hungary	JN417754 (Pintye et al. 2012)
Vitis115	*Erysiphe necator*	*Vitis vinifera*	22 Sept. 2009	Eger, Hungary	JN417756 (Pintye et al. 2012)
Vitis117	*Erysiphe necator*	*Vitis vinifera*	22 Sept. 2009	Eger, Hungary	JN417757 (Pintye et al. 2012)
G273 (ATCC200245)	*Erysiphe necator*	*Vitis* sp.	1989	Geneva, NY, USA	HM125018 (Kiss et al. 2011)
SF423 (ATCC200250)	*Erysiphe necator*	*Vitis riparia*	1991	Geneva, NY, USA	HM125017 (Kiss et al. 2011)
Ampelomyces* strains isolated from *Arthrocladiella mougeotii* infecting *Lycium halimfolium					
**A1 (ATCC201056)**	*Arthrocladiella mougeotii*	*Lycium halimfolium*	1990	Hungary	
A8	*Arthrocladiella mougeotii*	*Lycium halimfolium*	14 Sept. 2007	Site 1, bush 1, Budapest, Hungary	HM124894 (Kiss et al. 2011)
A10-a	*Arthrocladiella mougeotii*	*Lycium halimfolium*	14 Sept. 2007	Site 1, bush 1, Budapest, Hungary	HM124896 (Kiss et al. 2011)
A10-c	*Arthrocladiella mougeotii*	*Lycium halimfolium*	14 Sept. 2007	Site 1, bush 1, Budapest, Hungary	
A11-a	*Arthrocladiella mougeotii*	*Lycium halimfolium*	14 Sept. 2007	Site 1, bush 2, Budapest, Hungary	HM124897 (Kiss et al. 2011)
A11-b	*Arthrocladiella mougeotii*	*Lycium halimfolium*	14 Sept. 2007	Site 1, bush 2, Budapest, Hungary	
A11-c	*Arthrocladiella mougeotii*	*Lycium halimfolium*	14 Sept. 2007	Site 1, bush 2, Budapest, Hungary	
A12-a	*Arthrocladiella mougeotii*	*Lycium halimfolium*	19 Sept. 2007	Site 1, bush 1, Budapest, Hungary	HM124898 (Kiss et al. 2011)
A12-c	*Arthrocladiella mougeotii*	*Lycium halimfolium*	19 Sept. 2007	Site 1, bush 1, Budapest, Hungary	
A13-a	*Arthrocladiella mougeotii*	*Lycium halimfolium*	19 Sept. 2007	Site 1, bush 1, Budapest, Hungary	
A13-b	*Arthrocladiella mougeotii*	*Lycium halimfolium*	19 Sept. 2007	Site 1, bush 1, Budapest, Hungary	
A13-c	*Arthrocladiella mougeotii*	*Lycium halimfolium*	19 Sept. 2007	Site 1, bush 1, Budapest, Hungary	
A14-a	*Arthrocladiella mougeotii*	*Lycium halimfolium*	19 Sept. 2007	Site 1, bush 1, Budapest, Hungary	HM124899 (Kiss et al. 2011)
A14-b	*Arthrocladiella mougeotii*	*Lycium halimfolium*	19 Sept. 2007	Site 1, bush 1, Budapest, Hungary	
A14-c	*Arthrocladiella mougeotii*	*Lycium halimfolium*	19 Sept. 2007	Site 1, bush 1, Budapest, Hungary	
A14-d	*Arthrocladiella mougeotii*	*Lycium halimfolium*	19 Sept. 2007	Site 1, bush 1, Budapest, Hungary	
A15-a	*Arthrocladiella mougeotii*	*Lycium halimfolium*	21 Sept. 2007	Site 1, bush 1, Budapest, Hungary	HM124900 (Kiss et al. 2011)
A17	*Arthrocladiella mougeotii*	*Lycium halimfolium*	21 Sept. 2007	Site 1, bush 1, Budapest, Hungary	HM124901 (Kiss et al. 2011)
A18-a	*Arthrocladiella mougeotii*	*Lycium halimfolium*	14 Sept. 2007	Site 2, bush 1, Budapest, Hungary	HM124902 (Kiss et al. 2011)
A19-a	*Arthrocladiella mougeotii*	*Lycium halimfolium*	11 Oct. 2007	Site 2, bush 2, Budapest, Hungary	HM124903 (Kiss et al. 2011)
A19-b	*Arthrocladiella mougeotii*	*Lycium halimfolium*	11 Oct. 2007	Site 2, bush 2, Budapest, Hungary	
A19-c	*Arthrocladiella mougeotii*	*Lycium halimfolium*	11 Oct. 2007	Site 2, bush 2, Budapest, Hungary	
A20-a	*Arthrocladiella mougeotii*	*Lycium halimfolium*	11 Oct. 2007	Site 2, bush 2, Budapest, Hungary	HM124904 (Kiss et al. 2011)
A20-b	*Arthrocladiella mougeotii*	*Lycium halimfolium*	11 Oct. 2007	Site 2, bush 2, Budapest, Hungary	HM124905 (Kiss et al. 2011)
A20-c	*Arthrocladiella mougeotii*	*Lycium halimfolium*	11 Oct. 2007	Site 2, bush 2, Budapest, Hungary	
A26	*Arthrocladiella mougeotii*	*Lycium halimfolium*	23 Sept. 2007	Site 4, bush 2, Budapest, Hungary	HM124907 (Kiss et al. 2011)
A27-a	*Arthrocladiella mougeotii*	*Lycium halimfolium*	23 Sept. 2007	Site 4, bush 2, Budapest, Hungary	HM124908 (Kiss et al. 2011)
A27-b	*Arthrocladiella mougeotii*	*Lycium halimfolium*	23 Sept. 2007	Site 4, bush 2, Budapest, Hungary	

A33-c	*Arthrocladiella mougeotii*	*Lycium halimfolium*	23 Sept. 2007	Site 4, bush 3, Budapest, Hungary	
A33-e	*Arthrocladiella mougeotii*	*Lycium halimfolium*	23 Sept. 2007	Site 4, bush 3, Budapest, Hungary	HM124913 (Kiss et al. 2011)
A34-d	*Arthrocladiella mougeotii*	*Lycium halimfolium*	23 Sept. 2007	Site 4, bush 3, Budapest, Hungary	
A36-a	*Arthrocladiella mougeotii*	*Lycium halimfolium*	4 Oct. 2007	Site 5, bush 1, Budapest, Hungary	
A37-b	*Arthrocladiella mougeotii*	*Lycium halimfolium*	4 Oct. 2007	Site 5, bush 1, Budapest, Hungary	
A38-a	*Arthrocladiella mougeotii*	*Lycium halimfolium*	4 Oct. 2007	Site 5, bush 1, Budapest, Hungary	HM124915 (Kiss et al. 2011)
A38-b	*Arthrocladiella mougeotii*	*Lycium halimfolium*	4 Oct. 2007	Site 5, bush 1, Budapest, Hungary	
A38-c	*Arthrocladiella mougeotii*	*Lycium halimfolium*	4 Oct. 2007	Site 5, bush 1, Budapest, Hungary	
A39-a	*Arthrocladiella mougeotii*	*Lycium halimfolium*	4 Oct. 2007	Site 5, bush 2, Budapest, Hungary	HM124916 (Kiss et al. 2011)
A39-b	*Arthrocladiella mougeotii*	*Lycium halimfolium*	4 Oct. 2007	Site 5, bush 2, Budapest, Hungary	
A39-c	*Arthrocladiella mougeotii*	*Lycium halimfolium*	4 Oct. 2007	Site 5, bush 2, Budapest, Hungary	
A41-b	*Arthrocladiella mougeotii*	*Lycium halimfolium*	4 Oct. 2007	Site 5, bush 2, Budapest, Hungary	
A41-c	*Arthrocladiella mougeotii*	*Lycium halimfolium*	4 Oct. 2007	Site 5, bush 2, Budapest, Hungary	HM124918 (Kiss et al. 2011)
A45-a	*Arthrocladiella mougeotii*	*Lycium halimfolium*	4 Oct. 2007	Site 5, bush 3, Budapest, Hungary	HM124920 (Kiss et al. 2011)
A45-b	*Arthrocladiella mougeotii*	*Lycium halimfolium*	4 Oct. 2007	Site 5, bush 3, Budapest, Hungary	
A46-b	*Arthrocladiella mougeotii*	*Lycium halimfolium*	4 Oct. 2007	Site 5, bush 3, Budapest, Hungary	
A47-b	*Arthrocladiella mougeotii*	*Lycium halimfolium*	4 Oct. 2007	Site 5, bush 3, Budapest, Hungary	HM124921 (Kiss et al. 2011)
A49	*Arthrocladiella mougeotii*	*Lycium halimfolium*	4 Oct. 2007	Site 6, bush 1, Budapest, Hungary	
A51	*Arthrocladiella mougeotii*	*Lycium halimfolium*	4 Oct. 2007	Site 6, bush 1, Budapest, Hungary	
A52-b	*Arthrocladiella mougeotii*	*Lycium halimfolium*	4 Oct. 2007	Site 6, bush 1, Budapest, Hungary	
A53-a	*Arthrocladiella mougeotii*	*Lycium halimfolium*	5 Oct. 2007	Site 6, bush 2, Budapest, Hungary	
A53-b	*Arthrocladiella mougeotii*	*Lycium halimfolium*	5 Oct. 2007	Site 6, bush 2, Budapest, Hungary	
A53-c	*Arthrocladiella mougeotii*	*Lycium halimfolium*	5 Oct. 2007	Site 6, bush 2, Budapest, Hungary	
A54-a	*Arthrocladiella mougeotii*	*Lycium halimfolium*	5 Oct. 2007	Site 6, bush 2, Budapest, Hungary	
A54-b	*Arthrocladiella mougeotii*	*Lycium halimfolium*	5 Oct. 2007	Site 6, bush 2, Budapest, Hungary	
A54-c	*Arthrocladiella mougeotii*	*Lycium halimfolium*	5 Oct. 2007	Site 6, bush 2, Budapest, Hungary	
A55-a	*Arthrocladiella mougeotii*	*Lycium halimfolium*	5 Oct. 2007	Site 6, bush 2, Budapest, Hungary	
A55-b	*Arthrocladiella mougeotii*	*Lycium halimfolium*	5 Oct. 2007	Site 6, bush 2, Budapest, Hungary	
A55-c	*Arthrocladiella mougeotii*	*Lycium halimfolium*	5 Oct. 2007	Site 6, bush 2, Budapest, Hungary	
A57-a	*Arthrocladiella mougeotii*	*Lycium halimfolium*	30 Oct. 2007	Site 4, bush 4, Budapest, Hungary	
A57-b	*Arthrocladiella mougeotii*	*Lycium halimfolium*	30 Oct. 2007	Site 4, bush 4, Budapest, Hungary	
A58-b	*Arthrocladiella mougeotii*	*Lycium halimfolium*	30 Oct. 2007	Site 4, bush 5, Budapest, Hungary	
A59	*Arthrocladiella mougeotii*	*Lycium halimfolium*	30 Oct. 2007	Site 4, bush 5, Budapest, Hungary	
A60	*Arthrocladiella mougeotii*	*Lycium halimfolium*	1 Oct. 2007	Salföld, Hungary	
A61-a	*Arthrocladiella mougeotii*	*Lycium halimfolium*	2 Oct. 2007	Bush 1, Páty, Hungary	
A61-b	*Arthrocladiella mougeotii*	*Lycium halimfolium*	2 Oct. 2007	Bush 1, Páty, Hungary	HM124922 (Kiss et al. 2011)
A61-c	*Arthrocladiella mougeotii*	*Lycium halimfolium*	2 Oct. 2007	Bush 1, Páty, Hungary	
A62-a	*Arthrocladiella mougeotii*	*Lycium halimfolium*	2 Oct. 2007	Bush 1, Páty, Hungary	HM124923 (Kiss et al. 2011)

A62-b	*Arthrocladiella mougeotii*	*Lycium halimfolium*	2 Oct. 2007	Bush 1, Páty, Hungary	HM124924 (Kiss et al. 2011)
A62-c	*Arthrocladiella mougeotii*	*Lycium halimfolium*	2 Oct. 2007	Bush 1, Páty, Hungary	
A63-a	*Arthrocladiella mougeotii*	*Lycium halimfolium*	2 Oct. 2007	Bush 1, Páty, Hungary	HM124925 (Kiss et al. 2011)
A64-a	*Arthrocladiella mougeotii*	*Lycium halimfolium*	2 Oct. 2007	Bush 1, Páty, Hungary	
A64-b	*Arthrocladiella mougeotii*	*Lycium halimfolium*	2 Oct. 2007	Bush 1, Páty, Hungary	
A64-c	*Arthrocladiella mougeotii*	*Lycium halimfolium*	2 Oct. 2007	Bush 1, Páty, Hungary	
A69-a	*Arthrocladiella mougeotii*	*Lycium halimfolium*	2 Oct. 2007	Bush 2, Páty, Hungary	
A69-b	*Arthrocladiella mougeotii*	*Lycium halimfolium*	2 Oct. 2007	Bush 2, Páty, Hungary	
A70-a	*Arthrocladiella mougeotii*	*Lycium halimfolium*	2 Oct. 2007	Bush 2, Páty, Hungary	
A70-b	*Arthrocladiella mougeotii*	*Lycium halimfolium*	2 Oct. 2007	Bush 2, Páty, Hungary	
A71-a	*Arthrocladiella mougeotii*	*Lycium halimfolium*	2 Oct. 2007	Bush 2, Páty, Hungary	
A71-b	*Arthrocladiella mougeotii*	*Lycium halimfolium*	2 Oct. 2007	Bush 2, Páty, Hungary	
A72-a	*Arthrocladiella mougeotii*	*Lycium halimfolium*	2 Oct. 2007	Bush 2, Páty, Hungary	
A72-b	*Arthrocladiella mougeotii*	*Lycium halimfolium*	2 Oct. 2007	Bush 2, Páty, Hungary	
A72-c	*Arthrocladiella mougeotii*	*Lycium halimfolium*	2 Oct. 2007	Bush 2, Páty, Hungary	
A72-d	*Arthrocladiella mougeotii*	*Lycium halimfolium*	2 Oct. 2007	Bush 2, Páty, Hungary	
A75-a	*Arthrocladiella mougeotii*	*Lycium halimfolium*	2 Oct. 2007	Bush 2, Páty, Hungary	
A75-b	*Arthrocladiella mougeotii*	*Lycium halimfolium*	2 Oct. 2007	Bush 2, Páty, Hungary	
A75-c	*Arthrocladiella mougeotii*	*Lycium halimfolium*	2 Oct. 2007	Bush 2, Páty, Hungary	
A76-a	*Arthrocladiella mougeotii*	*Lycium halimfolium*	2 Oct. 2007	Bush 2, Páty, Hungary	
A76-b	*Arthrocladiella mougeotii*	*Lycium halimfolium*	2 Oct. 2007	Bush 2, Páty, Hungary	
A76-c	*Arthrocladiella mougeotii*	*Lycium halimfolium*	2 Oct. 2007	Bush 2, Páty, Hungary	
A77-a	*Arthrocladiella mougeotii*	*Lycium halimfolium*	2 Oct. 2007	Bush 2, Páty, Hungary	HM124927 (Kiss et al. 2011)
A77-b	*Arthrocladiella mougeotii*	*Lycium halimfolium*	2 Oct. 2007	Bush 2, Páty, Hungary	
A80-a	*Arthrocladiella mougeotii*	*Lycium halimfolium*	2 Oct. 2007	Bush 1, Budakeszi, Hungary	
A80-b	*Arthrocladiella mougeotii*	*Lycium halimfolium*	2 Oct. 2007	Bush 1, Budakeszi, Hungary	
A81-a	*Arthrocladiella mougeotii*	*Lycium halimfolium*	2 Oct. 2007	Bush 1, Budakeszi, Hungary	HM124930 (Kiss et al. 2011)
A82-a	*Arthrocladiella mougeotii*	*Lycium halimfolium*	4 Oct. 2007	Bush 2, Budakeszi, Hungary	HM124931 (Kiss et al. 2011)
A82-b	*Arthrocladiella mougeotii*	*Lycium halimfolium*	4 Oct. 2007	Bush 2, Budakeszi, Hungary	
A83	*Arthrocladiella mougeotii*	*Lycium halimfolium*	4 Oct. 2007	Bush 2, Budakeszi, Hungary	
A84-a	*Arthrocladiella mougeotii*	*Lycium halimfolium*	4 Oct. 2007	Bush 2, Budakeszi, Hungary	
A84-b	*Arthrocladiella mougeotii*	*Lycium halimfolium*	4 Oct. 2007	Bush 2, Budakeszi, Hungary	
A84-c	*Arthrocladiella mougeotii*	*Lycium halimfolium*	4 Oct. 2007	Bush 2, Budakeszi, Hungary	
A85	*Arthrocladiella mougeotii*	*Lycium halimfolium*	4 Oct. 2007	Bush 2, Budakeszi, Hungary	
A86-a	*Arthrocladiella mougeotii*	*Lycium halimfolium*	3 Oct. 2007	Bush 3, Budakeszi, Hungary	HM124932 (Kiss et al. 2011)
A86-b	*Arthrocladiella mougeotii*	*Lycium halimfolium*	4 Oct. 2007	Bush 3, Budakeszi, Hungary	
A86-c	*Arthrocladiella mougeotii*	*Lycium halimfolium*	4 Oct. 2007	Bush 3, Budakeszi, Hungary	
A86-d	*Arthrocladiella mougeotii*	*Lycium halimfolium*	4 Oct. 2007	Bush 3, Budakeszi, Hungary	

A86-e	*Arthrocladiella mougeotii*	*Lycium halimfolium*	4 Oct. 2007	Bush 3, Budakeszi, Hungary	
A87-a	*Arthrocladiella mougeotii*	*Lycium halimfolium*	4 Oct. 2007	Bush 3, Budakeszi, Hungary	
A87-b	*Arthrocladiella mougeotii*	*Lycium halimfolium*	4 Oct. 2007	Bush 3, Budakeszi, Hungary	
A87-c	*Arthrocladiella mougeotii*	*Lycium halimfolium*	4 Oct. 2007	Bush 3, Budakeszi, Hungary	HM124933 (Kiss et al. 2011)
A87-d	*Arthrocladiella mougeotii*	*Lycium halimfolium*	4 Oct. 2007	Bush 3, Budakeszi, Hungary	
A88-a	*Arthrocladiella mougeotii*	*Lycium halimfolium*	4 Oct. 2007	Bush 3, Budakeszi, Hungary	
A88-b	*Arthrocladiella mougeotii*	*Lycium halimfolium*	4 Oct. 2007	Bush 3, Budakeszi, Hungary	
A88-c	*Arthrocladiella mougeotii*	*Lycium halimfolium*	4 Oct. 2007	Bush 3, Budakeszi, Hungary	
A88-d	*Arthrocladiella mougeotii*	*Lycium halimfolium*	4 Oct. 2007	Bush 3, Budakeszi, Hungary	
A89-a	*Arthrocladiella mougeotii*	*Lycium halimfolium*	4 Oct. 2007	Bush 4, Budakeszi, Hungary	
A89-b	*Arthrocladiella mougeotii*	*Lycium halimfolium*	4 Oct. 2007	Bush 4, Budakeszi, Hungary	
A89-c	*Arthrocladiella mougeotii*	*Lycium halimfolium*	4 Oct. 2007	Bush 4, Budakeszi, Hungary	
A89-d	*Arthrocladiella mougeotii*	*Lycium halimfolium*	4 Oct. 2007	Bush 4, Budakeszi, Hungary	
A90-c	*Arthrocladiella mougeotii*	*Lycium halimfolium*	4 Oct. 2007	Bush 4, Budakeszi, Hungary	
A90-d	*Arthrocladiella mougeotii*	*Lycium halimfolium*	4 Oct. 2007	Bush 4, Budakeszi, Hungary	
A91-a	*Arthrocladiella mougeotii*	*Lycium halimfolium*	4 Oct. 2007	Bush 4, Budakeszi, Hungary	
A91-b	*Arthrocladiella mougeotii*	*Lycium halimfolium*	4 Oct. 2007	Bush 4, Budakeszi, Hungary	
A91-c	*Arthrocladiella mougeotii*	*Lycium halimfolium*	4 Oct. 2007	Bush 4, Budakeszi, Hungary	
A91-d	*Arthrocladiella mougeotii*	*Lycium halimfolium*	4 Oct. 2007	Bush 4, Budakeszi, Hungary	
A92-a	*Arthrocladiella mougeotii*	*Lycium halimfolium*	3 Oct. 2007	Bush 1, Biatorbágy, Hungary	
A92-b	*Arthrocladiella mougeotii*	*Lycium halimfolium*	3 Oct. 2007	Bush 1, Biatorbágy, Hungary	
A92-c	*Arthrocladiella mougeotii*	*Lycium halimfolium*	3 Oct. 2007	Bush 1, Biatorbágy, Hungary	HM124934 (Kiss et al. 2011)
A92-d	*Arthrocladiella mougeotii*	*Lycium halimfolium*	3 Oct. 2007	Bush 1, Biatorbágy, Hungary	
A92-e	*Arthrocladiella mougeotii*	*Lycium halimfolium*	3 Oct. 2007	Bush 1, Biatorbágy, Hungary	
A94-a	*Arthrocladiella mougeotii*	*Lycium halimfolium*	3 Oct. 2007	Bush 2, Biatorbágy, Hungary	HM124936 (Kiss et al. 2011)
A96-a	*Arthrocladiella mougeotii*	*Lycium halimfolium*	3 Oct. 2007	Bush 3, Biatorbágy, Hungary	
A96-b	*Arthrocladiella mougeotii*	*Lycium halimfolium*	3 Oct. 2007	Bush 3, Biatorbágy, Hungary	
A96-c	*Arthrocladiella mougeotii*	*Lycium halimfolium*	3 Oct. 2007	Bush 3, Biatorbágy, Hungary	HM124937 (Kiss et al. 2011)
A96-d	*Arthrocladiella mougeotii*	*Lycium halimfolium*	3 Oct. 2007	Bush 3, Biatorbágy, Hungary	
A97	*Arthrocladiella mougeotii*	*Lycium halimfolium*	3 Oct. 2007	Bush 3, Biatorbágy, Hungary	HM124938 (Kiss et al. 2011)
A99	*Arthrocladiella mougeotii*	*Lycium halimfolium*	3 Oct. 2007	Bush 3, Biatorbágy, Hungary	HM124940 (Kiss et al. 2011)
A101	*Arthrocladiella mougeotii*	*Lycium halimfolium*	3 Oct. 2007	Bush 4, Biatorbágy, Hungary	
A102-a	*Arthrocladiella mougeotii*	*Lycium halimfolium*	3 Oct. 2007	Bush 4, Biatorbágy, Hungary	
A102-b	*Arthrocladiella mougeotii*	*Lycium halimfolium*	3 Oct. 2007	Bush 4, Biatorbágy, Hungary	
A102-c	*Arthrocladiella mougeotii*	*Lycium halimfolium*	3 Oct. 2007	Bush 4, Biatorbágy, Hungary	
A103	*Arthrocladiella mougeotii*	*Lycium halimfolium*	3 Oct. 2007	Bush 5, Biatorbágy, Hungary	
A104-a	*Arthrocladiella mougeotii*	*Lycium halimfolium*	3 Oct. 2007	Bush 5, Biatorbágy, Hungary	HM124941 (Kiss et al. 2011)
A104-b	*Arthrocladiella mougeotii*	*Lycium halimfolium*	3 Oct. 2007	Bush 5, Biatorbágy, Hungary	

A104-c	*Arthrocladiella mougeotii*	*Lycium halimfolium*	3 Oct. 2007	Bush 5, Biatorbágy, Hungary	
A105	*Arthrocladiella mougeotii*	*Lycium halimfolium*	3 Oct. 2007	Bush 5, Biatorbágy, Hungary	HM124942 (Kiss et al. 2011)
A108-a	*Arthrocladiella mougeotii*	*Lycium halimfolium*	18 Sept. 2007	Site 8, Budapest, Hungary	HM124944 (Kiss et al. 2011)
A108-b	*Arthrocladiella mougeotii*	*Lycium halimfolium*	18 Sept. 2007	Site 8, Budapest, Hungary	
A108-d	*Arthrocladiella mougeotii*	*Lycium halimfolium*	18 Sept. 2007	Site 8, Budapest, Hungary	
A109-e	*Arthrocladiella mougeotii*	*Lycium halimfolium*	18 Sept. 2007	Site 8, Budapest, Hungary	
A110-b	*Arthrocladiella mougeotii*	*Lycium halimfolium*	18 Sept. 2007	Site 8, Budapest, Hungary	HM124947 (Kiss et al. 2011)
A110-c	*Arthrocladiella mougeotii*	*Lycium halimfolium*	18 Sept. 2007	Site 8, Budapest, Hungary	
A110-d	*Arthrocladiella mougeotii*	*Lycium halimfolium*	18 Sept. 2007	Site 8, Budapest, Hungary	
A111-a	*Arthrocladiella mougeotii*	*Lycium halimfolium*	18 Sept. 2007	Site 8, Budapest, Hungary	HM124948 (Kiss et al. 2011)
A111-b	*Arthrocladiella mougeotii*	*Lycium halimfolium*	18 Sept. 2007	Site 8, Budapest, Hungary	HM124949 (Kiss et al. 2011)
A113-a	*Arthrocladiella mougeotii*	*Lycium halimfolium*	18 Sept. 2007	Site 8, Budapest, Hungary	HM124951 (Kiss et al. 2011)
A113-b	*Arthrocladiella mougeotii*	*Lycium halimfolium*	18 Sept. 2007	Site 8, Budapest, Hungary	HM124952 (Kiss et al. 2011)
**A115**	*Arthrocladiella mougeotii*	*Lycium halimfolium*	18 Sept. 2007	Site 8, Budapest, Hungary	HM124955 (Kiss et al. 2011)
A120	*Arthrocladiella mougeotii*	*Lycium halimfolium*	2 Oct. 2007	Bush 1, Páty, Hungary	
Ampelomyces strains isolated from apple powdery mildew (Podosphaera leucotricha)					
B5	*Podosphaera leucotricha*	*Malus domestica*	May 2000	Keszű, Hungary	
B6	*Podosphaera leucotricha*	*Malus domestica*	April 2001	Budapest, Hungary	
B15	*Podosphaera leucotricha*	*Malus domestica*	April 2001	Keszű, Hungary	
B26	*Podosphaera leucotricha*	*Malus domestica*	May 2002	Dresden, Germany	
B33 (MYA-3395)	*Podosphaera leucotricha*	*Malus domestica*	May 2002	Ahrensburg, Germany	AY663817 (Szentiványi et al. 2005)
B34 (MYA-3396)	*Podosphaera leucotricha*	*Malus domestica*	May 2002	Cambridge, UK	AY663818 (Szentiványi et al. 2005)
B37	*Podosphaera leucotricha*	*Malus domestica*	May 2002	Canterbury, UK	
B38	*Podosphaera leucotricha*	*Malus domestica*	May 2002	Canterbury, UK	
B41	*Podosphaera leucotricha*	*Malus domestica*	May 2002	Canterbury, UK	
B42	*Podosphaera leucotricha*	*Malus domestica*	May 2002	East Malling, UK	
B46	*Podosphaera leucotricha*	*Malus domestica*	May 2002	East Malling, UK	
B58	*Podosphaera leucotricha*	*Malus robusta*	9 April 2008	Tree 1, Orsay, France	
B59	*Podosphaera leucotricha*	*Malus robusta*	9 April 2008	Tree 1, Orsay, France	
B60	*Podosphaera leucotricha*	*Malus robusta*	9 April 2008	Tree 1, Orsay, France	
B61	*Podosphaera leucotricha*	*Malus robusta*	9 April 2008	Tree 2, Orsay, France	HM124960 (Kiss et al. 2011)
B62	*Podosphaera leucotricha*	*Malus robusta*	9 April 2008	Tree 3, Orsay, France	
B63	*Podosphaera leucotricha*	*Malus robusta*	9 April 2008	Tree 3, Orsay, France	
B64	*Podosphaera leucotricha*	*Malus robusta*	9 April 2008	Tree 3, Orsay, France	
B65	*Podosphaera leucotricha*	*Malus robusta*	9 April 2008	Tree 3, Orsay, France	
B66	*Podosphaera leucotricha*	*Malus domestica*	3 May 2008	Site 1, Tree 1, Cambridge, UK	
B67	*Podosphaera leucotricha*	*Malus domestica*	3 May 2008	Site 1, Tree 1, Cambridge, UK	

B68	*Podosphaera leucotricha*	*Malus domestica*	3 May 2008	Site 1, Tree 1, Cambridge, UK	
B69	*Podosphaera leucotricha*	*Malus domestica*	3 May 2008	Site 1, Tree 1, Cambridge, UK	
B70	*Podosphaera leucotricha*	*Malus domestica*	3 May 2008	Site 1, Tree 1, Cambridge, UK	
B71	*Podosphaera leucotricha*	*Malus domestica*	3 May 2008	Site 1, Tree 1, Cambridge, UK	
B72	*Podosphaera leucotricha*	*Malus domestica*	3 May 2008	Site 1, Tree 1, Cambridge, UK	
B74	*Podosphaera leucotricha*	*Malus domestica*	3 May 2008	Site 1, Tree 1, Cambridge, UK	
B75	*Podosphaera leucotricha*	*Malus domestica*	3 May 2008	Site 1, Tree 1, Cambridge, UK	
B76	*Podosphaera leucotricha*	*Malus domestica*	4 May 2008	Site 1, Tree 2, Cambridge, UK	
B77	*Podosphaera leucotricha*	*Malus domestica*	4 May 2008	Site 1, Tree 2, Cambridge, UK	
B79	*Podosphaera leucotricha*	*Malus domestica*	5 May 2008	Stansted, UK	
B80	*Podosphaera leucotricha*	*Malus domestica*	5 May 2008	Stansted, UK	
B82	*Podosphaera leucotricha*	*Malus domestica*	5 May 2008	Stansted, UK	HM124961 (Kiss et al. 2011)
B83	*Podosphaera leucotricha*	*Malus domestica*	5 May 2008	Stansted, UK	
B84	*Podosphaera leucotricha*	*Malus domestica*	5 May 2008	Stansted, UK	
B85	*Podosphaera leucotricha*	*Malus domestica*	5 May 2008	Stansted, UK	
B86	*Podosphaera leucotricha*	*Malus domestica*	5 May 2008	Stansted, UK	
B87	*Podosphaera leucotricha*	*Malus domestica*	7 May 2008	Site 2, Cambridge, UK	
B89	*Podosphaera leucotricha*	*Malus domestica*	7 May 2008	Site 2, Cambridge, UK	
B90	*Podosphaera leucotricha*	*Malus domestica*	7 May 2008	Site 2, Cambridge, UK	
B91	*Podosphaera leucotricha*	*Malus domestica*	8 May 2008	Tree 1, Canterbury, UK	
B92	*Podosphaera leucotricha*	*Malus domestica*	8 May 2008	Tree 1, Canterbury, UK	
B94	*Podosphaera leucotricha*	*Malus domestica*	8 May 2008	Tree 1, Canterbury, UK	HM124962 (Kiss et al. 2011)
B95	*Podosphaera leucotricha*	*Malus domestica*	8 May 2008	Tree 1, Canterbury, UK	
B97	*Podosphaera leucotricha*	*Malus domestica*	8 May 2008	Tree 2, Canterbury, UK	
B99	*Podosphaera leucotricha*	*Malus domestica*	8 May 2008	Tree 2, Canterbury, UK	
B100	*Podosphaera leucotricha*	*Malus domestica*	8 May 2008	Tree 2, Canterbury, UK	
B101	*Podosphaera leucotricha*	*Malus mandshurica*	9 May 2008	Site 1, East Malling, UK	
B103	*Podosphaera leucotricha*	*Malus brevipes*	9 May 2008	Site 1, East Malling, UK	
B105	*Podosphaera leucotricha*	*Malus domestica*	9 May 2008	Site 2, East Malling, UK	
B106	*Podosphaera leucotricha*	*Malus domestica*	9 May 2008	Site 2, East Malling, UK	
B107	*Podosphaera leucotricha*	*Malus domestica*	9 May 2008	Site 2, East Malling, UK	
B108	*Podosphaera leucotricha*	*Malus domestica*	9 May 2008	Site 2, East Malling, UK	
B109	*Podosphaera leucotricha*	*Malus domestica*	8 May 2008	Whittlesford, UK	
B110	*Podosphaera leucotricha*	*Malus domestica*	8 May 2008	Whittlesford, UK	
B111	*Podosphaera leucotricha*	*Malus domestica*	8 May 2008	Whittlesford, UK	
B114	*Podosphaera leucotricha*	*Malus domestica*	26 May 2008	Biatorbágy, Hungary	
B115	*Podosphaera leucotricha*	*Malus domestica*	26 May 2008	Biatorbágy, Hungary	
B116	*Podosphaera leucotricha*	*Malus domestica*	1 June 2008	Velem, Hungary	

B118	*Podosphaera leucotricha*	*Malus domestica*	28 April 2008	Tree 1, Eperjeske, Hungary	HM124963 (Kiss et al. 2011)
B119-a	*Podosphaera leucotricha*	*Malus domestica*	28 April 2008	Tree 1, Eperjeske, Hungary	
B119-b	*Podosphaera leucotricha*	*Malus domestica*	28 April 2008	Tree 1, Eperjeske, Hungary	
B120-b	*Podosphaera leucotricha*	*Malus domestica*	28 April 2008	Tree 1, Eperjeske, Hungary	
B121-a	*Podosphaera leucotricha*	*Malus domestica*	28 April 2008	Tree 1, Eperjeske, Hungary	
B121-b	*Podosphaera leucotricha*	*Malus domestica*	28 April 2008	Tree 1, Eperjeske, Hungary	
B122-a	*Podosphaera leucotricha*	*Malus domestica*	28 April 2008	Tree 1, Eperjeske, Hungary	
B122-b	*Podosphaera leucotricha*	*Malus domestica*	28 April 2008	Tree 1, Eperjeske, Hungary	
B123-a	*Podosphaera leucotricha*	*Malus domestica*	28 April 2008	Tree 2, Eperjeske, Hungary	
B124-a	*Podosphaera leucotricha*	*Malus domestica*	28 April 2008	Tree 2, Eperjeske, Hungary	HM124964 (Kiss et al. 2011)
B124-b	*Podosphaera leucotricha*	*Malus domestica*	28 April 2008	Tree 2, Eperjeske, Hungary	
B125-a	*Podosphaera leucotricha*	*Malus domestica*	28 April 2008	Tree 2, Eperjeske, Hungary	
B125-b	*Podosphaera leucotricha*	*Malus domestica*	28 April 2008	Tree 2, Eperjeske, Hungary	
B126-a	*Podosphaera leucotricha*	*Malus domestica*	28 April 2008	Tree 3, Eperjeske, Hungary	
B126-b	*Podosphaera leucotricha*	*Malus domestica*	28 April 2008	Tree 3, Eperjeske, Hungary	
B127-a	*Podosphaera leucotricha*	*Malus domestica*	28 April 2008	Tree 4, Eperjeske, Hungary	
B127-b	*Podosphaera leucotricha*	*Malus domestica*	28 April 2008	Tree 4, Eperjeske, Hungary	
B128-a	*Podosphaera leucotricha*	*Malus domestica*	28 April 2008	Tree 5, Eperjeske, Hungary	
B128-b	*Podosphaera leucotricha*	*Malus domestica*	28 April 2008	Tree 5, Eperjeske, Hungary	
B129	*Podosphaera leucotricha*	*Malus domestica*	28 April 2008	Tree 5, Eperjeske, Hungary	
B130-a	*Podosphaera leucotricha*	*Malus domestica*	28 April 2008	Tree 5, Eperjeske, Hungary	
B130-b	*Podosphaera leucotricha*	*Malus domestica*	28 April 2008	Tree 5, Eperjeske, Hungary	
B131	*Podosphaera leucotricha*	*Malus domestica*	28 April 2008	Tree 5, Eperjeske, Hungary	
B132-a	*Podosphaera leucotricha*	*Malus domestica*	28 April 2008	Tree 5, Eperjeske, Hungary	
B132-b	*Podosphaera leucotricha*	*Malus domestica*	28 April 2008	Tree 5, Eperjeske, Hungary	
B133	*Podosphaera leucotricha*	*Malus domestica*	28 April 2008	Tree 5, Eperjeske, Hungary	
B135	*Podosphaera leucotricha*	*Malus domestica*	28 April 2008	Tree 5, Eperjeske, Hungary	
B136-a	*Podosphaera leucotricha*	*Malus domestica*	28 April 2008	Tree 5, Eperjeske, Hungary	
B136-b	*Podosphaera leucotricha*	*Malus domestica*	28 April 2008	Tree 5, Eperjeske, Hungary	
B137	*Podosphaera leucotricha*	*Malus domestica*	22 April 2008	Site 9, Tree 1, Budapest, Hungary	
B138-a	*Podosphaera leucotricha*	*Malus domestica*	22 April 2008	Site 9, Tree 1, Budapest, Hungary	
B138-b	*Podosphaera leucotricha*	*Malus domestica*	22 April 2008	Site 9, Tree 1, Budapest, Hungary	
B140-a	*Podosphaera leucotricha*	*Malus domestica*	22 April 2008	Site 9, Tree 1, Budapest, Hungary	
B140-b	*Podosphaera leucotricha*	*Malus domestica*	22 April 2008	Site 9, Tree 1, Budapest, Hungary	
B141-a	*Podosphaera leucotricha*	*Malus domestica*	22 April 2008	Site 9, Tree 1, Budapest, Hungary	
B141-b	*Podosphaera leucotricha*	*Malus domestica*	22 April 2008	Site 9, Tree 1, Budapest, Hungary	
B141-c	*Podosphaera leucotricha*	*Malus domestica*	22 April 2008	Site 9, Tree 1, Budapest, Hungary	
B141-d	*Podosphaera leucotricha*	*Malus domestica*	22 April 2008	Site 9, Tree 1, Budapest, Hungary	

B141-e	*Podosphaera leucotricha*	*Malus domestica*	22 April 2008	Site 9, Tree 1, Budapest, Hungary
B143	*Podosphaera leucotricha*	*Malus domestica*	22 April 2008	Site 9, Tree 1, Budapest, Hungary
B144-a	*Podosphaera leucotricha*	*Malus domestica*	22 April 2008	Site 9, Tree 1, Budapest, Hungary
B144-b	*Podosphaera leucotricha*	*Malus domestica*	22 April 2008	Site 9, Tree 1, Budapest, Hungary
B146	*Podosphaera leucotricha*	*Malus domestica*	22 April 2008	Site 9, Tree 2, Budapest, Hungary
B147	*Podosphaera leucotricha*	*Malus domestica*	22 April 2008	Site 9, Tree 2, Budapest, Hungary
B148	*Podosphaera leucotricha*	*Malus domestica*	22 April 2008	Site 9, Tree 2, Budapest, Hungary
B149-a	*Podosphaera leucotricha*	*Malus domestica*	22 April 2008	Site 9, Tree 2, Budapest, Hungary
B149-b	*Podosphaera leucotricha*	*Malus domestica*	22 April 2008	Site 9, Tree 2, Budapest, Hungary
B150-a	*Podosphaera leucotricha*	*Malus domestica*	22 April 2008	Site 9, Tree 3, Budapest, Hungary
B150-b	*Podosphaera leucotricha*	*Malus domestica*	22 April 2008	Site 9, Tree 3, Budapest, Hungary
B150-c	*Podosphaera leucotricha*	*Malus domestica*	22 April 2008	Site 9, Tree 3, Budapest, Hungary
B151-a	*Podosphaera leucotricha*	*Malus domestica*	22 April 2008	Site 9, Tree 3, Budapest, Hungary
B151-b	*Podosphaera leucotricha*	*Malus domestica*	22 April 2008	Site 9, Tree 3, Budapest, Hungary
B151-c	*Podosphaera leucotricha*	*Malus domestica*	22 April 2008	Site 9, Tree 3, Budapest, Hungary
B152-a	*Podosphaera leucotricha*	*Malus domestica*	22 April 2008	Site 9, Tree 3, Budapest, Hungary
B152-b	*Podosphaera leucotricha*	*Malus domestica*	22 April 2008	Site 9, Tree 3, Budapest, Hungary
B153-a	*Podosphaera leucotricha*	*Malus domestica*	15 May 2008	Site 10, Budapest, Hungary
B153-b	*Podosphaera leucotricha*	*Malus domestica*	15 May 2008	Site 10, Budapest, Hungary
B154	*Podosphaera leucotricha*	*Malus domestica*	15 May 2008	Site 10, Budapest, Hungary
B155	*Podosphaera leucotricha*	*Malus domestica*	15 May 2008	Site 10, Budapest, Hungary
B156-b	*Podosphaera leucotricha*	*Malus domestica*	15 May 2008	Site 10, Budapest, Hungary
B157-a	*Podosphaera leucotricha*	*Malus domestica*	15 May 2008	Site 10, Budapest, Hungary
B157-b	*Podosphaera leucotricha*	*Malus domestica*	15 May 2008	Site 10, Budapest, Hungary
B158-a	*Podosphaera leucotricha*	*Malus domestica*	15 May 2008	Site 10, Budapest, Hungary
B158-b	*Podosphaera leucotricha*	*Malus domestica*	15 May 2008	Site 10, Budapest, Hungary
B158-c	*Podosphaera leucotricha*	*Malus domestica*	15 May 2008	Site 10, Budapest, Hungary
B158-d	*Podosphaera leucotricha*	*Malus domestica*	15 May 2008	Site 10, Budapest, Hungary
B159-a	*Podosphaera leucotricha*	*Malus domestica*	15 May 2008	Site 10, Budapest, Hungary
B159-b	*Podosphaera leucotricha*	*Malus domestica*	15 May 2008	Site 10, Budapest, Hungary
B160-b	*Podosphaera leucotricha*	*Malus domestica*	15 May 2008	Site 10, Budapest, Hungary
B161	*Podosphaera leucotricha*	*Malus domestica*	15 May 2008	Site 10, Budapest, Hungary
B162-b	*Podosphaera leucotricha*	*Malus domestica*	15 May 2008	Site 10, Budapest, Hungary
B163-a	*Podosphaera leucotricha*	*Malus domestica*	15 May 2008	Site 10, Budapest, Hungary
B163-b	*Podosphaera leucotricha*	*Malus domestica*	15 May 2008	Site 10, Budapest, Hungary
B164-a	*Podosphaera leucotricha*	*Malus domestica*	15 May 2008	Site 10, Budapest, Hungary
B164-b	*Podosphaera leucotricha*	*Malus domestica*	15 May 2008	Site 10, Budapest, Hungary
B165	*Podosphaera leucotricha*	*Malus domestica*	15 May 2008	Site 10, Budapest, Hungary

B167-a	*Podosphaera leucotricha*	*Malus domestica*	15 May 2008	Site 10, Budapest, Hungary	
B167-b	*Podosphaera leucotricha*	*Malus domestica*	15 May 2008	Site 10, Budapest, Hungary	
B168	*Podosphaera leucotricha*	*Malus domestica*	15 May 2008	Site 10, Budapest, Hungary	
B169-b	*Podosphaera leucotricha*	*Malus domestica*	15 May 2008	Site 10, Budapest, Hungary	
B170-a	*Podosphaera leucotricha*	*Malus domestica*	15 May 2008	Site 10, Budapest, Hungary	
B171-b	*Podosphaera leucotricha*	*Malus domestica*	15 May 2008	Site 10, Budapest, Hungary	
B172-a	*Podosphaera leucotricha*	*Malus domestica*	15 May 2008	Site 10, Budapest, Hungary	
B175	*Podosphaera leucotricha*	*Malus domestica*	15 May 2008	Site 10, Budapest, Hungary	
B176	*Podosphaera leucotricha*	*Malus domestica*	15 May 2008	Site 10, Budapest, Hungary	HM124966 (Kiss et al. 2011)
B177-b	*Podosphaera leucotricha*	*Malus domestica*	15 May 2008	Site 10, Budapest, Hungary	
B179-a	*Podosphaera leucotricha*	*Malus domestica*	15 May 2008	Site 10, Budapest, Hungary	
B179-b	*Podosphaera leucotricha*	*Malus domestica*	15 May 2008	Site 10, Budapest, Hungary	
B180-b	*Podosphaera leucotricha*	*Malus domestica*	15 May 2008	Site 10, Budapest, Hungary	
B181-b	*Podosphaera leucotricha*	*Malus domestica*	15 May 2008	Site 10, Budapest, Hungary	
B182	*Podosphaera leucotricha*	*Malus domestica*	30 April 2008	Gödöllő, Hungary	
B183	*Podosphaera leucotricha*	*Malus domestica*	30 April 2008	Gödöllő, Hungary	
B184-b	*Podosphaera leucotricha*	*Malus domestica*	30 April 2008	Gödöllő, Hungary	
B185	*Podosphaera leucotricha*	*Malus domestica*	1 June 2008	Keszű, Hungary	
B186-a	*Podosphaera leucotricha*	*Malus domestica*	1 June 2008	Keszű, Hungary	
B186-b	*Podosphaera leucotricha*	*Malus domestica*	1 June 2008	Keszű, Hungary	
B187-a	*Podosphaera leucotricha*	*Malus domestica*	1 June 2008	Keszű, Hungary	
B187-b	*Podosphaera leucotricha*	*Malus domestica*	1 June 2008	Keszű, Hungary	
B188	*Podosphaera leucotricha*	*Malus domestica*	1 June 2008	Keszű, Hungary	
B189	*Podosphaera leucotricha*	*Malus domestica*	1 June 2008	Keszű, Hungary	
B190	*Podosphaera leucotricha*	*Malus domestica*	1 June 2008	Keszű, Hungary	HM124967 (Kiss et al. 2011)
B191-a	*Podosphaera leucotricha*	*Malus domestica*	1 June 2008	Keszű, Hungary	
B191-b	*Podosphaera leucotricha*	*Malus domestica*	1 June 2008	Keszű, Hungary	
B192	*Podosphaera leucotricha*	*Malus domestica*	2 May 2008	Tree 1, Orsay, France	
B193	*Podosphaera leucotricha*	*Malus domestica*	2 May 2008	Tree 9, Orsay, France	
B194	*Podosphaera leucotricha*	*Malus domestica*	2 May 2008	Tree 11, Orsay, France	
B195	*Podosphaera leucotricha*	*Malus domestica*	2 May 2008	Tree 12, Orsay, France	
B196	*Podosphaera leucotricha*	*Malus domestica*	2 May 2008	Tree 14, Orsay, France	
B197	*Podosphaera leucotricha*	*Malus domestica*	2 May 2008	Tree 14, Orsay, France	
B198	*Podosphaera leucotricha*	*Malus domestica*	2 May 2008	Tree 14, Orsay, France	
B199	*Podosphaera leucotricha*	*Malus domestica*	2 May 2008	Tree 15, Orsay, France	
B200	*Podosphaera leucotricha*	*Malus domestica*	2 May 2008	Tree 15, Orsay, France	HM124968 (Kiss et al. 2011)
B201	*Podosphaera leucotricha*	*Malus domestica*	2 May 2008	Tree 16, Orsay, France	
B202	*Podosphaera leucotricha*	*Malus domestica*	2 May 2008	Tree 18, Orsay, France	

B203	*Podosphaera leucotricha*	*Malus domestica*	2 May 2008	Tree 20, Orsay, France	
B204	*Podosphaera leucotricha*	*Malus domestica*	2 May 2008	Tree 21, Orsay, France	
B205	*Podosphaera leucotricha*	*Malus domestica*	2 May 2008	Tree 23, Orsay, France	
B206	*Podosphaera leucotricha*	*Malus domestica*	2 May 2008	Tree 23, Orsay, France	
B207-a	*Podosphaera leucotricha*	*Malus domestica*	5 May 2008	Site 1, tree 1, Gotheron, France	
B207-b	*Podosphaera leucotricha*	*Malus domestica*	5 May 2008	Site 1, tree 1, Gotheron, France	
B209-a	*Podosphaera leucotricha*	*Malus domestica*	5 May 2008	Site 1, tree 1, Gotheron, France	
B209-b	*Podosphaera leucotricha*	*Malus domestica*	5 May 2008	Site 1, tree 1, Gotheron, France	
B210	*Podosphaera leucotricha*	*Malus domestica*	5 May 2008	Site 1, tree 1, Gotheron, France	
B211	*Podosphaera leucotricha*	*Malus domestica*	5 May 2008	Site 1, tree 2, Gotheron, France	
B212-a	*Podosphaera leucotricha*	*Malus domestica*	5 May 2008	Site 1, tree 2, Gotheron, France	
B212-b	*Podosphaera leucotricha*	*Malus domestica*	5 May 2008	Site 1, tree 2, Gotheron, France	
B213	*Podosphaera leucotricha*	*Malus domestica*	5 May 2008	Site 1, tree 2, Gotheron, France	
B214-a	*Podosphaera leucotricha*	*Malus domestica*	5 May 2008	Site 1, tree 3, Gotheron, France	
B214-b	*Podosphaera leucotricha*	*Malus domestica*	5 May 2008	Site 1, tree 3, Gotheron, France	
B215	*Podosphaera leucotricha*	*Malus domestica*	5 May 2008	Site 1, tree 3, Gotheron, France	
B216	*Podosphaera leucotricha*	*Malus domestica*	5 May 2008	Site 1, tree 3, Gotheron, France	
B217-a	*Podosphaera leucotricha*	*Malus domestica*	5 May 2008	Site 1, tree 4, Gotheron, France	
B217-b	*Podosphaera leucotricha*	*Malus domestica*	5 May 2008	Site 1, tree 4, Gotheron, France	
B218-a	*Podosphaera leucotricha*	*Malus domestica*	5 May 2008	Site 1, tree 5, Gotheron, France	
B218-b	*Podosphaera leucotricha*	*Malus domestica*	5 May 2008	Site 1, tree 5, Gotheron, France	
B219-a	*Podosphaera leucotricha*	*Malus domestica*	5 May 2008	Site 1, tree 5, Gotheron, France	
B219-b	*Podosphaera leucotricha*	*Malus domestica*	5 May 2008	Site 1, tree 5, Gotheron, France	
B219-c	*Podosphaera leucotricha*	*Malus domestica*	5 May 2008	Site 1, tree 5, Gotheron, France	
B220-a	*Podosphaera leucotricha*	*Malus domestica*	5 May 2008	Site 1, tree 5, Gotheron, France	
B220-b	*Podosphaera leucotricha*	*Malus domestica*	5 May 2008	Site 1, tree 5, Gotheron, France	
B221	*Podosphaera leucotricha*	*Malus domestica*	5 May 2008	Site 1, tree 6, Gotheron, France	HM124969 (Kiss et al. 2011)
B222-a	*Podosphaera leucotricha*	*Malus domestica*	5 May 2008	Site 1, tree 6, Gotheron, France	
B222-b	*Podosphaera leucotricha*	*Malus domestica*	5 May 2008	Site 1, tree 6, Gotheron, France	
B223-a	*Podosphaera leucotricha*	*Malus domestica*	5 May 2008	Site 1, tree 6, Gotheron, France	
B223-b	*Podosphaera leucotricha*	*Malus domestica*	5 May 2008	Site 1, tree 6, Gotheron, France	
B224-a	*Podosphaera leucotricha*	*Malus domestica*	5 May 2008	Site 1, tree 7, Gotheron, France	
B225-a	*Podosphaera leucotricha*	*Malus domestica*	5 May 2008	Site 1, tree 7, Gotheron, France	
B225-b	*Podosphaera leucotricha*	*Malus domestica*	5 May 2008	Site 1, tree 7, Gotheron, France	
B226-a	*Podosphaera leucotricha*	*Malus domestica*	5 May 2008	Site 1, tree 8, Gotheron, France	
B226-b	*Podosphaera leucotricha*	*Malus domestica*	5 May 2008	Site 1, tree 8, Gotheron, France	
B227-a	*Podosphaera leucotricha*	*Malus domestica*	5 May 2008	Site 1, tree 9, Gotheron, France	
B227-b	*Podosphaera leucotricha*	*Malus domestica*	5 May 2008	Site 1, tree 9, Gotheron, France	

B228	*Podosphaera leucotricha*	*Malus domestica*	5 May 2008	Site 1, tree 9, Gotheron, France
B229-a	*Podosphaera leucotricha*	*Malus domestica*	5 May 2008	Site 1, tree 10, Gotheron, France
B229-b	*Podosphaera leucotricha*	*Malus domestica*	5 May 2008	Site 1, tree 10, Gotheron, France
B230-a	*Podosphaera leucotricha*	*Malus domestica*	5 May 2008	Site 1, tree 11, Gotheron, France
B230-b	*Podosphaera leucotricha*	*Malus domestica*	5 May 2008	Site 1, tree 11, Gotheron, France
B231	*Podosphaera leucotricha*	*Malus domestica*	5 May 2008	Site 1, tree 11, Gotheron, France
B234	*Podosphaera leucotricha*	*Malus domestica*	5 May 2008	Site 1, tree 13, Gotheron, France
B235	*Podosphaera leucotricha*	*Malus domestica*	5 May 2008	Site 1, tree 13, Gotheron, France
B236-a	*Podosphaera leucotricha*	*Malus domestica*	5 May 2008	Site 1, tree 14, Gotheron, France
B236-b	*Podosphaera leucotricha*	*Malus domestica*	5 May 2008	Site 1, tree 14, Gotheron, France
B237-a	*Podosphaera leucotricha*	*Malus domestica*	5 May 2008	Site 1, tree 14, Gotheron, France
B237-b	*Podosphaera leucotricha*	*Malus domestica*	5 May 2008	Site 1, tree 14, Gotheron, France
B239-a	*Podosphaera leucotricha*	*Malus domestica*	5 May 2008	Site 1, tree 15, Gotheron, France
B239-b	*Podosphaera leucotricha*	*Malus domestica*	5 May 2008	Site 1, tree 15, Gotheron, France
B240-a	*Podosphaera leucotricha*	*Malus domestica*	6 May 2008	Site 2, tree 1, Gotheron, France
B240-b	*Podosphaera leucotricha*	*Malus domestica*	6 May 2008	Site 2, tree 1, Gotheron, France
B242	*Podosphaera leucotricha*	*Malus domestica*	6 May 2008	Site 2, tree 5, Gotheron, France
B244-a	*Podosphaera leucotricha*	*Malus domestica*	6 May 2008	Site 2, tree 7, Gotheron, France
B244-b	*Podosphaera leucotricha*	*Malus domestica*	6 May 2008	Site 2, tree 7, Gotheron, France
B245	*Podosphaera leucotricha*	*Malus domestica*	17 Sept. 2007	Eperjeske, Hungary
B246	*Podosphaera leucotricha*	*Malus domestica*	28 April 2008	Tree 2, Eperjeske, Hungary

Powdery mildew-infected apple leaf samples with *Ampelomyces*

b13	*Podosphaera leucotricha*	*Malus robusta*	9 April 2008	Tree 1, Orsay, France
b20	*Podosphaera leucotricha*	*Malus robusta*	9 April 2008	Tree 2, Orsay, France
b25	*Podosphaera leucotricha*	*Malus domestica*	2 May 2008	Tree 2, Orsay, France
b26	*Podosphaera leucotricha*	*Malus domestica*	2 May 2008	Tree 2, Orsay, France
b27	*Podosphaera leucotricha*	*Malus domestica*	2 May 2008	Tree 3, Orsay, France
b30	*Podosphaera leucotricha*	*Malus domestica*	2 May 2008	Tree 3, Orsay, France
b31	*Podosphaera leucotricha*	*Malus domestica*	2 May 2008	Tree 3, Orsay, France
b33	*Podosphaera leucotricha*	*Malus domestica*	2 May 2008	Tree 3, Orsay, France
b34	*Podosphaera leucotricha*	*Malus domestica*	2 May 2008	Tree 3, Orsay, France
b35	*Podosphaera leucotricha*	*Malus domestica*	2 May 2008	Tree 3, Orsay, France
b36	*Podosphaera leucotricha*	*Malus domestica*	2 May 2008	Tree 3, Orsay, France
b41	*Podosphaera leucotricha*	*Malus domestica*	2 May 2008	Tree 4, Orsay, France
b44	*Podosphaera leucotricha*	*Malus domestica*	2 May 2008	Tree 4, Orsay, France
b46	*Podosphaera leucotricha*	*Malus domestica*	2 May 2008	Tree 4, Orsay, France
b49	*Podosphaera leucotricha*	*Malus domestica*	2 May 2008	Tree 4, Orsay, France

b51	*Podosphaera leucotricha*	*Malus domestica*	2 May 2008	Tree 5, Orsay, France
b52	*Podosphaera leucotricha*	*Malus domestica*	2 May 2008	Tree 6, Orsay, France
b55	*Podosphaera leucotricha*	*Malus domestica*	2 May 2008	Tree 8, Orsay, France
b58	*Podosphaera leucotricha*	*Malus domestica*	2 May 2008	Tree 9, Orsay, France
b59	*Podosphaera leucotricha*	*Malus domestica*	2 May 2008	Tree 9, Orsay, France
b60	*Podosphaera leucotricha*	*Malus domestica*	2 May 2008	Tree 10, Orsay, France
b62	*Podosphaera leucotricha*	*Malus domestica*	2 May 2008	Tree 11, Orsay, France
b65	*Podosphaera leucotricha*	*Malus domestica*	2 May 2008	Tree 13, Orsay, France
b66	*Podosphaera leucotricha*	*Malus domestica*	2 May 2008	Tree 13, Orsay, France
b69	*Podosphaera leucotricha*	*Malus domestica*	2 May 2008	Tree 14, Orsay, France
b74	*Podosphaera leucotricha*	*Malus domestica*	2 May 2008	Tree 14, Orsay, France
b76	*Podosphaera leucotricha*	*Malus domestica*	2 May 2008	Tree 14, Orsay, France
b79	*Podosphaera leucotricha*	*Malus domestica*	2 May 2008	Tree 14, Orsay, France
b81	*Podosphaera leucotricha*	*Malus domestica*	2 May 2008	Tree 14, Orsay, France
b85	*Podosphaera leucotricha*	*Malus domestica*	2 May 2008	Tree 17, Orsay, France
b87	*Podosphaera leucotricha*	*Malus domestica*	2 May 2008	Tree 17, Orsay, France
b88	*Podosphaera leucotricha*	*Malus domestica*	2 May 2008	Tree 17, Orsay, France
b92	*Podosphaera leucotricha*	*Malus domestica*	2 May 2008	Tree 18, Orsay, France
b94	*Podosphaera leucotricha*	*Malus domestica*	2 May 2008	Tree 18, Orsay, France
b96	*Podosphaera leucotricha*	*Malus domestica*	2 May 2008	Tree 18, Orsay, France
b98	*Podosphaera leucotricha*	*Malus domestica*	2 May 2008	Tree 18, Orsay, France
b99	*Podosphaera leucotricha*	*Malus domestica*	2 May 2008	Tree 19, Orsay, France
b100	*Podosphaera leucotricha*	*Malus domestica*	2 May 2008	Tree 19, Orsay, France
b102	*Podosphaera leucotricha*	*Malus domestica*	2 May 2008	Tree 19, Orsay, France
b107	*Podosphaera leucotricha*	*Malus domestica*	2 May 2008	Tree 19, Orsay, France
b110	*Podosphaera leucotricha*	*Malus domestica*	2 May 2008	Tree 19, Orsay, France
b111	*Podosphaera leucotricha*	*Malus domestica*	2 May 2008	Tree 21, Orsay, France
b113	*Podosphaera leucotricha*	*Malus domestica*	2 May 2008	Tree 22, Orsay, France
b117	*Podosphaera leucotricha*	*Malus domestica*	2 May 2008	Tree 22, Orsay, France
b119	*Podosphaera leucotricha*	*Malus domestica*	2 May 2008	Tree 22, Orsay, France
b121	*Podosphaera leucotricha*	*Malus domestica*	2 May 2008	Tree 22, Orsay, France
b122	*Podosphaera leucotricha*	*Malus domestica*	2 May 2008	Tree 22, Orsay, France
b123	*Podosphaera leucotricha*	*Malus domestica*	2 May 2008	Tree 22, Orsay, France
b124	*Podosphaera leucotricha*	*Malus domestica*	2 May 2008	Tree 22, Orsay, France
b125	*Podosphaera leucotricha*	*Malus domestica*	2 May 2008	Tree 22, Orsay, France
b128	*Podosphaera leucotricha*	*Malus domestica*	2 May 2008	Tree 22, Orsay, France
b129	*Podosphaera leucotricha*	*Malus domestica*	2 May 2008	Tree 22, Orsay, France
b130	*Podosphaera leucotricha*	*Malus domestica*	2 May 2008	Tree 22, Orsay, France

b131	*Podosphaera leucotricha*	*Malus domestica*	2 May 2008	Tree 22, Orsay, France
b132	*Podosphaera leucotricha*	*Malus domestica*	2 May 2008	Tree 22, Orsay, France
b133	*Podosphaera leucotricha*	*Malus domestica*	2 May 2008	Tree 22, Orsay, France
b134	*Podosphaera leucotricha*	*Malus domestica*	2 May 2008	Tree 22, Orsay, France
b137	*Podosphaera leucotricha*	*Malus domestica*	2 May 2008	Tree 22, Orsay, France
b139	*Podosphaera leucotricha*	*Malus domestica*	2 May 2008	Tree 22, Orsay, France
b140	*Podosphaera leucotricha*	*Malus domestica*	2 May 2008	Tree 22, Orsay, France
b143	*Podosphaera leucotricha*	*Malus domestica*	2 May 2008	Tree 23, Orsay, France
b144	*Podosphaera leucotricha*	*Malus domestica*	2 May 2008	Tree 23, Orsay, France
b146	*Podosphaera leucotricha*	*Malus domestica*	2 May 2008	Tree 24, Orsay, France
b147	*Podosphaera leucotricha*	*Malus domestica*	2 May 2008	Tree 25, Orsay, France
b153	*Podosphaera leucotricha*	*Malus domestica*	2 May 2008	Tree 26, Orsay, France
b154	*Podosphaera leucotricha*	*Malus domestica*	2 May 2008	Tree 26, Orsay, France
b155	*Podosphaera leucotricha*	*Malus domestica*	2 May 2008	Tree 27, Orsay, France
b157	*Podosphaera leucotricha*	*Malus domestica*	2 May 2008	Tree 27, Orsay, France
b164	*Podosphaera leucotricha*	*Malus domestica*	2 May 2008	Tree 27, Orsay, France
b166	*Podosphaera leucotricha*	*Malus domestica*	2 May 2008	Tree 27, Orsay, France
b167	*Podosphaera leucotricha*	*Malus domestica*	2 May 2008	Tree 27, Orsay, France
b169	*Podosphaera leucotricha*	*Malus domestica*	5 May 2008	Site 1, tree 1, Gotheron, France
b170	*Podosphaera leucotricha*	*Malus domestica*	5 May 2008	Site 1, tree 1, Gotheron, France
b171	*Podosphaera leucotricha*	*Malus domestica*	5 May 2008	Site 1, tree 1, Gotheron, France
b174	*Podosphaera leucotricha*	*Malus domestica*	5 May 2008	Site 1, tree 1, Gotheron, France
b175	*Podosphaera leucotricha*	*Malus domestica*	5 May 2008	Site 1, tree 1, Gotheron, France
b177	*Podosphaera leucotricha*	*Malus domestica*	5 May 2008	Site 1, tree 1, Gotheron, France
b178	*Podosphaera leucotricha*	*Malus domestica*	5 May 2008	Site 1, tree 1, Gotheron, France
b182	*Podosphaera leucotricha*	*Malus domestica*	5 May 2008	Site 1, tree 2, Gotheron, France
b183	*Podosphaera leucotricha*	*Malus domestica*	5 May 2008	Site 1, tree 2, Gotheron, France
b185	*Podosphaera leucotricha*	*Malus domestica*	5 May 2008	Site 1, tree 3, Gotheron, France
b186	*Podosphaera leucotricha*	*Malus domestica*	5 May 2008	Site 1, tree 3, Gotheron, France
b190	*Podosphaera leucotricha*	*Malus domestica*	5 May 2008	Site 1, tree 3, Gotheron, France
b192	*Podosphaera leucotricha*	*Malus domestica*	5 May 2008	Site 1, tree 3, Gotheron, France
b193	*Podosphaera leucotricha*	*Malus domestica*	5 May 2008	Site 1, tree 3, Gotheron, France
b195	*Podosphaera leucotricha*	*Malus domestica*	5 May 2008	Site 1, tree 3, Gotheron, France
b196	*Podosphaera leucotricha*	*Malus domestica*	5 May 2008	Site 1, tree 3, Gotheron, France
b197	*Podosphaera leucotricha*	*Malus domestica*	5 May 2008	Site 1, tree 3, Gotheron, France
b199	*Podosphaera leucotricha*	*Malus domestica*	5 May 2008	Site 1, tree 3, Gotheron, France
b201	*Podosphaera leucotricha*	*Malus domestica*	5 May 2008	Site 1, tree 3, Gotheron, France
b205	*Podosphaera leucotricha*	*Malus domestica*	5 May 2008	Site 1, tree 3, Gotheron, France

b206	*Podosphaera leucotricha*	*Malus domestica*	5 May 2008	Site 1, tree 3, Gotheron, France
b207	*Podosphaera leucotricha*	*Malus domestica*	5 May 2008	Site 1, tree 3, Gotheron, France
b209	*Podosphaera leucotricha*	*Malus domestica*	5 May 2008	Site 1, tree 3, Gotheron, France
b210	*Podosphaera leucotricha*	*Malus domestica*	5 May 2008	Site 1, tree 3, Gotheron, France
b211	*Podosphaera leucotricha*	*Malus domestica*	5 May 2008	Site 1, tree 3, Gotheron, France
b212	*Podosphaera leucotricha*	*Malus domestica*	5 May 2008	Site 1, tree 5, Gotheron, France
b217	*Podosphaera leucotricha*	*Malus domestica*	5 May 2008	Site 1, tree 6, Gotheron, France
b218	*Podosphaera leucotricha*	*Malus domestica*	5 May 2008	Site 1, tree 6, Gotheron, France
b219	*Podosphaera leucotricha*	*Malus domestica*	5 May 2008	Site 1, tree 6, Gotheron, France
b220	*Podosphaera leucotricha*	*Malus domestica*	5 May 2008	Site 1, tree 7, Gotheron, France
b222	*Podosphaera leucotricha*	*Malus domestica*	5 May 2008	Site 1, tree 7, Gotheron, France
b223	*Podosphaera leucotricha*	*Malus domestica*	5 May 2008	Site 1, tree 8, Gotheron, France
b225	*Podosphaera leucotricha*	*Malus domestica*	5 May 2008	Site 1, tree 10, Gotheron, France
b226	*Podosphaera leucotricha*	*Malus domestica*	5 May 2008	Site 1, tree 12, Gotheron, France
b227	*Podosphaera leucotricha*	*Malus domestica*	5 May 2008	Site 1, tree 13, Gotheron, France
b228	*Podosphaera leucotricha*	*Malus domestica*	5 May 2008	Site 1, tree 13, Gotheron, France
b229	*Podosphaera leucotricha*	*Malus domestica*	5 May 2008	Site 1, tree 14, Gotheron, France
b230	*Podosphaera leucotricha*	*Malus domestica*	5 May 2008	Site 1, tree 14, Gotheron, France
b231	*Podosphaera leucotricha*	*Malus domestica*	5 May 2008	Site 1, tree 14, Gotheron, France
b232	*Podosphaera leucotricha*	*Malus domestica*	5 May 2008	Site 1, tree 14, Gotheron, France
b233	*Podosphaera leucotricha*	*Malus domestica*	6 May 2008	Site 2, tree 1, Gotheron, France
b234	*Podosphaera leucotricha*	*Malus domestica*	6 May 2008	Site 2, tree 1, Gotheron, France
b235	*Podosphaera leucotricha*	*Malus domestica*	6 May 2008	Site 2, tree 1, Gotheron, France
b236	*Podosphaera leucotricha*	*Malus domestica*	6 May 2008	Site 2, tree 1, Gotheron, France
b237	*Podosphaera leucotricha*	*Malus domestica*	6 May 2008	Site 2, tree 1, Gotheron, France
b238	*Podosphaera leucotricha*	*Malus domestica*	6 May 2008	Site 2, tree 1, Gotheron, France
b241	*Podosphaera leucotricha*	*Malus domestica*	6 May 2008	Site 2, tree 3, Gotheron, France
b242	*Podosphaera leucotricha*	*Malus domestica*	6 May 2008	Site 2, tree 3, Gotheron, France
b243	*Podosphaera leucotricha*	*Malus domestica*	6 May 2008	Site 2, tree 3, Gotheron, France
b244	*Podosphaera leucotricha*	*Malus domestica*	6 May 2008	Site 2, tree 3, Gotheron, France
b245	*Podosphaera leucotricha*	*Malus domestica*	6 May 2008	Site 2, tree 3, Gotheron, France
b246	*Podosphaera leucotricha*	*Malus domestica*	6 May 2008	Site 2, tree 3, Gotheron, France
b248	*Podosphaera leucotricha*	*Malus domestica*	6 May 2008	Site 2, tree 4, Gotheron, France
b249	*Podosphaera leucotricha*	*Malus domestica*	6 May 2008	Site 2, tree 5, Gotheron, France
b250	*Podosphaera leucotricha*	*Malus domestica*	6 May 2008	Site 2, tree 5, Gotheron, France
b251	*Podosphaera leucotricha*	*Malus domestica*	6 May 2008	Site 2, tree 5, Gotheron, France
b252	*Podosphaera leucotricha*	*Malus domestica*	6 May 2008	Site 2, tree 5, Gotheron, France
b253	*Podosphaera leucotricha*	*Malus domestica*	6 May 2008	Site 2, tree 5, Gotheron, France

b256	*Podosphaera leucotricha*	*Malus domestica*	6 May 2008	Site 2, tree 5, Gotheron, France	
b257	*Podosphaera leucotricha*	*Malus domestica*	6 May 2008	Site 2, tree 5, Gotheron, France	
b258	*Podosphaera leucotricha*	*Malus domestica*	6 May 2008	Site 2, tree 5, Gotheron, France	
b261	*Podosphaera leucotricha*	*Malus domestica*	6 May 2008	Site 2, tree 5, Gotheron, France	
b263	*Podosphaera leucotricha*	*Malus domestica*	6 May 2008	Site 2, tree 6, Gotheron, France	
b266	*Podosphaera leucotricha*	*Malus domestica*	6 May 2008	Site 2, tree 7, Gotheron, France	
b267	*Podosphaera leucotricha*	*Malus domestica*	6 May 2008	Site 2, tree 7, Gotheron, France	
b268	*Podosphaera leucotricha*	*Malus domestica*	6 May 2008	Site 2, tree 7, Gotheron, France	
b269	*Podosphaera leucotricha*	*Malus domestica*	6 May 2008	Site 2, tree 7, Gotheron, France	
b271	*Podosphaera leucotricha*	*Malus domestica*	6 May 2008	Site 2, tree 7, Gotheron, France	
b273	*Podosphaera leucotricha*	*Malus domestica*	6 May 2008	Site 2, tree 8, Gotheron, France	
b275	*Podosphaera leucotricha*	*Malus domestica*	6 May 2008	Site 2, tree 8, Gotheron, France	
b282-a	*Podosphaera leucotricha*	*Malus domestica*	28 April 2008	Tree 6, Eperjeske, Hungary	
b285-a	*Podosphaera leucotricha*	*Malus domestica*	28 April 2008	Tree 6, Eperjeske, Hungary	
b287-a	*Podosphaera leucotricha*	*Malus domestica*	28 April 2008	Tree 6, Eperjeske, Hungary	
b288-c	*Podosphaera leucotricha*	*Malus domestica*	28 April 2008	Tree 6, Eperjeske, Hungary	
b291	*Podosphaera leucotricha*	*Malus domestica*	22 April 2008	Site 9, Tree 1, Budapest, Hungary	
b297	*Podosphaera leucotricha*	*Malus domestica*	30 April 2008	Gödöllő, Hungary	
b299	*Podosphaera leucotricha*	*Malus domestica*	30 April 2008	Gödöllő, Hungary	
b301	*Podosphaera leucotricha*	*Malus domestica*	30 April 2008	Gödöllő, Hungary	
b304	*Podosphaera leucotricha*	*Malus domestica*	30 April 2008	Gödöllő, Hungary	
b307	*Podosphaera leucotricha*	*Malus domestica*	30 April 2008	Gödöllő, Hungary	
b310	*Podosphaera leucotricha*	*Malus domestica*	30 April 2008	Gödöllő, Hungary	
b315	*Podosphaera leucotricha*	*Malus domestica*	June 2008	Keszű, Hungary	
b318	*Podosphaera leucotricha*	*Malus domestica*	June 2008	Keszű, Hungary	
b324	*Podosphaera leucotricha*	*Malus domestica*	June 2008	Keszű, Hungary	
b326	*Podosphaera leucotricha*	*Malus domestica*	June 2008	Keszű, Hungary	
b331	*Podosphaera leucotricha*	*Malus domestica*	June 2008	Keszű, Hungary	
b333	*Podosphaera leucotricha*	*Malus domestica*	June 2008	Keszű, Hungary	
b339	*Podosphaera leucotricha*	*Malus domestica*	June 2008	Keszű, Hungary	
b342	*Podosphaera leucotricha*	*Malus domestica*	June 2008	Velem, Hungary	
b344	*Podosphaera leucotricha*	*Malus domestica*	June 2008	Velem, Hungary	
b346	*Podosphaera leucotricha*	*Malus domestica*	June 2008	Velem, Hungary	
***Ampelomyces* strains isolated from other powdery mildew species**					
BV2	*Erysiphe berberidis*	*Berberis vulgaris*	20 Sept. 2007	Site 11, Budapest, Hungary	HM124958 (Kiss et al. 2011)
BV4-b	*Erysiphe berberidis*	*Berberis vulgaris*	20 Sept. 2007	Site 11, Budapest, Hungary	

BV4-c	*Erysiphe berberidis*	*Berberis vulgaris*	20 Sept. 2007	Site 11, Budapest, Hungary	
RA1-b	*Erysiphe polygoni*	*Rumex acetosa*	10 Oct. 2007	Site 1, Budapest, Hungary	
RA2-a	*Erysiphe polygoni*	*Rumex acetosa*	10 Oct. 2007	Site 1, Budapest, Hungary	HM125004 (Kiss et al. 2011)
RA2-d	*Erysiphe polygoni*	*Rumex acetosa*	10 Oct. 2007	Site 1, Budapest, Hungary	HM125005 (Kiss et al. 2011)
**MA1-c**	*Erysiphe berberidis*	*Mahonia aquifolium*	25 Sept. 2007	Site 12, Budapest, Hungary	HM124992 (Kiss et al. 2011)
MA5	*Erysiphe berberidis*	*Mahonia aquifolium*	11 Oct. 2007	Site 2, Budapest, Hungary	
MA6-b	*Erysiphe berberidis*	*Mahonia aquifolium*	11 Oct. 2007	Site 2, Budapest, Hungary	HM124996 (Kiss et al. 2011)
MA7-a	*Erysiphe berberidis*	*Mahonia aquifolium*	11 Oct. 2007	Site 2, Budapest, Hungary	
MA7-b	*Erysiphe berberidis*	*Mahonia aquifolium*	11 Oct. 2007	Site 2, Budapest, Hungary	
MA8	*Erysiphe berberidis*	*Mahonia aquifolium*	25 Sept. 2007	Site 12, Budapest, Hungary	HM124997 (Kiss et al. 2011)
MA-H	*Erysiphe berberidis*	*Mahonia aquifolium*	11 Oct. 2007	Site 2, Budapest, Hungary	
**RS1-a**	*Podosphaera pannosa*	*Rosa* sp.	5 Oct. 2007	Site 13, Budapest, Hungary	HM125010 (Kiss et al. 2011)
RS2-a	*Podosphaera pannosa*	*Rosa* sp.	5 Oct. 2007	Site 13, Budapest, Hungary	
RS2-b	*Podosphaera pannosa*	*Rosa* sp.	5 Oct. 2007	Site 13, Budapest, Hungary	
RS3-a	*Podosphaera pannosa*	*Rosa* sp.	5 Oct. 2007	Site 13, Budapest, Hungary	HM125011 (Kiss et al. 2011)
RS3-b	*Podosphaera pannosa*	*Rosa* sp.	5 Oct. 2007	Site 13, Budapest, Hungary	
PN1-a	*Erysiphe sordida*	*Plantago major*	9 Oct. 2007	Site 10, Budapest, Hungary	HM125000 (Kiss et al. 2011)
PN2	*Erysiphe sordida*	*Plantago major*	9 Oct. 2007	Site 10, Budapest, Hungary	
PN3	*Erysiphe sordida*	*Plantago major*	9 Oct. 2007	Site 10, Budapest, Hungary	
PN4-a	*Erysiphe sordida*	*Plantago major*	9 Oct. 2007	Site 10, Budapest, Hungary	
PN4-b	*Erysiphe sordida*	*Plantago major*	9 Oct. 2007	Site 10, Budapest, Hungary	HM125001 (Kiss et al. 2011)
XL1-a	*Erysiphe depressa*	*Arctium lappa*	10 Oct. 2007	Site 1, Budapest, Hungary	HM125025 (Kiss et al. 2011)
XL1-b	*Erysiphe depressa*	*Arctium lappa*	10 Oct. 2007	Site 1, Budapest, Hungary	
XL2-b	*Erysiphe depressa*	*Arctium lappa*	10 Oct. 2007	Site 1, Budapest, Hungary	HM125027 (Kiss et al. 2011)
XL3-a	*Erysiphe depressa*	*Arctium lappa*	10 Oct. 2007	Site 1, Budapest, Hungary	HM125028 (Kiss et al. 2011)
XL3-b	*Erysiphe depressa*	*Arctium lappa*	10 Oct. 2007	Site 1, Budapest, Hungary	HM125029 (Kiss et al. 2011)
XL4-a	*Erysiphe depressa*	*Arctium lappa*	10 Oct. 2007	Site 1, Budapest, Hungary	
XL4-b	*Erysiphe depressa*	*Arctium lappa*	10 Oct. 2007	Site 1, Budapest, Hungary	HM125030 (Kiss et al. 2011)
XL4-c	*Erysiphe depressa*	*Arctium lappa*	10 Oct. 2007	Site 1, Budapest, Hungary	
**LV2-b**	*Erysiphe* sp.	*Ligustrum vulgare*	17 Oct. 2007	Budakeszi, Hungary	HM124990 (Kiss et al. 2011)
TP1	*Erysiphe trifolii*	*Trifolium pratense*	3 Oct. 2007	Site 1, tree, Gotheron, France	HM125019 (Kiss et al. 2011)
TP3	*Erysiphe trifolii*	*Trifolium pratense*	3 Oct. 2007	Site 1, Gotheron, France	
TP4	*Erysiphe trifolii*	*Trifolium pratense*	3 Oct. 2007	Site 1, Gotheron, France	
TP5	*Erysiphe trifolii*	*Trifolium pratense*	3 Oct. 2007	Site 1, Gotheron, France	
TR1	*Erysiphe trifolii*	*Trifolium pratense*	3 Oct. 2007	Site 1, Gotheron, France	HM125024 (Kiss et al. 2011)
**H14**	*Erysiphe trifolii*	*Trifolium pratense*	3 Oct. 2007	Site 1, tree, Gotheron, France	
GS1	*Erysiphe cruciferarum*	*Alyssum calycinum*	5 May 2008	Site 1, Gotheron, France	HM124979 (Kiss et al. 2011)
GY-a	*Golovinomyces cichoracearum*	*Lactuca* sp.	5 May 2008	Site 1, Gotheron, France	HM124981 (Kiss et al. 2011)

GY-b	*Golovinomyces cichoracearum*	*Lactuca* sp.	5 May 2008	Site 1, Gotheron, France	HM124982 (Kiss et al. 2011)
GY-c	*Golovinomyces cichoracearum*	*Lactuca* sp.	5 May 2008	Site 1, Gotheron, France	
GL	*Neoerysiphe galeopsidis*	*Lamium* sp.	5 May 2008	Site 1, Gotheron, France	HM124978 (Kiss et al. 2011)
ALA1-a	*Golovinomyces cichoracearum*	*Lactuca* sp.	9 May 2008	Avignon, France	HM124956 (Kiss et al. 2011)
Aq SA	*Podosphaera pannosa*	*Rosa* sp.		Rep. South Africa	HM125014 (Kiss et al. 2011)
**HMLAC226**	*Erysiphe polygoni*	*Polygonum aviculare*	2003	Mengyin, Shandong, China	DQ490766 (Liang et al. 2007)
**G2**	*Erysiphe polygoni*	*Rumex patientia*	2002	Budapest, Hungary	DQ490770 (Liang et al. 2007)
**DSM 2222**	*Podosphaera xanthii*	*Cucumis* sp.		Germany	U82450 (Kiss & Nakasone 1998)
**AQ10**	*Oidium* sp.	*Catha edulis*		Israel	AF035783 (Kiss & Nakasone 1998)
GYER	*Erysiphe arcuata*	*Carpinus betulus*	Oct. 2008	Budapest, Hungary	HM124983 (Kiss et al 2011)
GW	*Erysiphe cynoglossi*	*Echium* sp.	4 May 2008	Site 1, Gotheron, France	HM124983 (Kiss et al. 2011)

Powdery mildew-infected leaf samples, other than apple leaves, containing *Ampelomyces* pycnidia

tp2	*Erysiphe trifolii*	*Trifolium pratense*	3 Oct. 2007	Site 1, Gotheron, France
tp13	*Erysiphe trifolii*	*Trifolium pratense*	3 Oct. 2007	Site 1, Gotheron, France
tp20	*Erysiphe trifolii*	*Trifolium pratense*	3 Oct. 2007	Site 1, Gotheron, France
tp21	*Erysiphe trifolii*	*Trifolium pratense*	3 Oct. 2007	Site 1, Gotheron, France
tp24	*Erysiphe trifolii*	*Trifolium pratense*	3 Oct. 2007	Site 1, Gotheron, France
tp25	*Erysiphe trifolii*	*Trifolium pratense*	3 Oct. 2007	Site 1, Gotheron, France
