# Supplementary material for: Host Phenology and Geography as Drivers of Differentiation in Generalist Fungal Mycoparasites
Source: PLoS One. 2015 Mar 24;10(3):e0120703. doi: 10.1371/journal.pone.0120703 (PMC4372539; doi:10.1371/journal.pone.0120703)
Supplement: S2 Table — (PDF) [file pone.0120703.s006.pdf]

Table S2: genotypes of *Ampelomyces* strains isolated from different mycohosts, genotyped with five microsatellite markers

	**Lk7c**	**Lk3a**	**Lk10c**	**Lk3b**	**Lk10d**
Ampelomyces* strains isolated from grass powdery mildew (*Blume					
BgrA	194	269	179	132	239
BgrB	194	269	179	132	239
BgrC	194	269	179	132	239
BgrD	194	269	179	132	239
BgrE	194	269	179	132	239
BgrF	194	269	179	132	239
BgrG	194	269	179	132	239
BgrH	194	269	179	132	239
BgrI	194	296	149	132	200
KACC 43563	176	269	179	132	239
KACC 44850	176	296	182	132	203
KACC 44851	194	269	179	132	239
***Ampelomyces* strains isolated from grapevine powdery mildew (*Erysiphe necator*)**					
Vitis42	194	242	176	132	239
Vitis55	194	242	179	132	251
Vitis56	194	242	179	132	230
Vitis60	194	254	179	132	239
Vitis70 (CBS 132	179	206	179	126	224
Vitis71	179	206	179	126	224
Vitis72	194	272	173	132	248
Vitis76	194	242	179	132	239
Vitis79	194	242	179	132	239
Vitis81	194	254	179	132	239
Vitis98	212	242	176	132	224
Vitis107	194	254	179	132	236
Vitis113	176	236	176	132	188
Vitis115	176	242	176	138	206
Vitis117	176	242	176	138	209
G273 (ATCC200	176	260	176	132	188
SF423 (ATCC20	176	236	176	132	188
Ampelomyces* strains isolated from *Arthrocladiella mougeotii* infecting *Lycium halimfolium					
A1 (ATCC20105	212	242	179	132	212
A8	203	263	176	132	221
A10-a	212	263	179	132	221
A10-c	212	263	179	132	221
A11-a	203	263	176	132	221
A11-b	203	263	176	132	221
A11-c	203	263	176	132	221
A12-a	203	263	176	132	221
A12-c	203	263	176	132	221
A13-a	203	263	176	132	221
A13-b	203	263	176	132	221
A13-c	203	263	176	132	221
A14-a	203	263	176	132	221

A14-b	203	263	176	132	221
A14-c	203	263	176	132	221
A14-d	203	263	176	132	221
A15-a	212	257	179	132	221
A17	212	257	179	132	221
A18-a	203	242	176	132	221
A19-a	212	242	176	132	212
A19-b	212	242	176	132	212
A19-c	212	242	176	132	212
A20-a	212	242	176	132	212
A20-b	212	242	176	132	212
A20-c	212	242	176	132	212
A26	212	263	179	132	212
A27-a	203	242	179	132	224
A27-b	203	242	179	132	224
A33-c	203	263	179	132	239
A33-e	203	263	179	132	239
A34-d	203	263	179	132	239
A36-a	188	209	197	126	221
A37-b	188	209	197	126	221
A38-a	188	209	197	126	221
A38-b	188	209	197	126	221
A38-c	188	209	197	126	221
A39-a	194	257	176	132	212
A39-b	188	209	197	126	215
A39-c	212	263	176	132	233
A41-b	194	257	176	132	212
A41-c	203	257	176	132	212
A45-a	188	209	197	126	221
A45-b	188	209	197	126	221
A46-b	188	209	197	126	221
A47-b	179	209	179	126	221
A49	203	242	179	132	221
A51	203	242	179	132	221
A52-b	194	248	179	132	224
A53-a	194	212	149	132	209
A53-b	194	212	149	132	209
A53-c	194	212	149	132	209
A54-a	194	212	149	132	209
A54-b	194	212	149	132	209
A54-c	194	212	149	132	209
A55-a	194	212	149	132	209
A55-b	194	212	149	132	209
A55-c	194	212	149	132	209
A57-a	194	242	179	132	239
A57-b	194	242	179	132	239
A58-b	194	254	179	132	257
A59	194	239	176	132	236
A60	194	269	179	132	239
A61-a	194	269	179	132	239

A61-b	194	269	179	132	239
A61-c	194	269	179	132	239
A62-a	194	269	179	132	239
A62-b	194	269	179	132	239
A62-c	194	269	179	132	239
A63-a	179	209	179	126	221
A64-a	194	269	179	132	239
A64-b	194	269	179	132	239
A64-c	194	269	179	132	239
A69-a	212	263	179	132	212
A69-b	212	263	179	132	212
A70-a	212	263	179	132	212
A70-b	212	263	179	132	212
A71-a	212	263	179	132	212
A71-b	212	263	179	132	212
A72-a	212	263	179	132	212
A72-b	212	263	179	132	212
A72-c	212	263	179	132	212
A72-d	212	263	179	132	212
A75-a	212	263	179	132	212
A75-b	212	263	179	132	212
A75-c	212	263	179	132	212
A76-a	212	263	179	132	212
A76-b	212	263	179	132	212
A76-c	212	263	179	132	212
A77-a	212	263	179	132	212
A77-b	212	263	179	132	212
A80-a	179	206	197	126	221
A80-b	179	206	179	126	221
A81-a	203	254	176	132	224
A82-a	212	242	179	132	224
A82-b	212	242	179	132	224
A83	212	257	179	132	224
A84-a	203	242	176	132	212
A84-b	212	248	176	132	221
A84-c	212	248	176	132	224
A85	203	242	176	132	224
A86-a	212	257	179	132	224
A86-b	179	206	197	126	221
A86-c	194	257	176	132	224
A86-d	212	242	176	132	212
A86-e	212	242	173	132	224
A87-a	221	242	179	132	230
A87-b	221	242	179	132	230
A87-c	194	257	176	132	224
A87-d	221	242	179	132	230
A88-a	212	242	179	132	224
A88-b	212	257	179	132	224
A88-c	212	242	176	132	212
A88-d	212	242	179	132	224

A89-a	212	242	173	132	224
A89-b	212	242	173	132	224
A89-c	212	257	179	132	224
A89-d	212	242	173	132	224
A90-c	212	242	176	132	212
A90-d	212	242	176	132	212
A91-a	212	242	173	132	224
A91-b	212	242	173	132	224
A91-c	194	257	176	132	224
A91-d	212	242	173	132	224
A92-a	194	254	179	132	242
A92-b	194	254	179	132	242
A92-c	194	254	179	132	242
A92-d	194	254	179	132	242
A92-e	194	254	179	132	242
A94-a	194	272	179	132	239
A96-a	194	272	179	132	239
A96-b	194	272	179	132	239
A96-c	194	272	179	132	239
A96-d	194	272	179	132	239
A97	194	242	176	132	239
A99	188	206	176	126	221
A101	194	242	176	132	212
A102-a	212	257	176	132	224
A102-b	212	257	176	132	224
A102-c	212	257	176	132	224
A103	194	272	179	132	239
A104-a	185	209	149	132	215
A104-b	212	257	176	132	224
A104-c	212	257	176	132	224
A105	185	209	149	132	215
A108-a	212	254	176	132	212
A108-b	212	254	176	132	212
A108-d	212	254	176	132	212
A109-e	188	206	197	126	221
A110-b	188	206	176	126	221
A110-c	212	269	179	132	224
A110-d	212	269	179	132	224
A111-a	212	269	179	132	224
A111-b	188	206	179	126	221
A113-a	212	269	179	132	224
A113-b	212	269	179	132	224
A115	212	254	176	132	212
A120	194	269	179	132	239

Ampelomyces* strains isolated from apple powdery mildew *(Podospharea leucotricha)

B5	176	242	176	138	206
B6	176	242	176	138	206
B15	176	242	176	138	206
B26	176	242	176	138	206

B33 (MYA-3395)	176	242	176	138	206
B34 (MYA-3396)	176	242	176	138	206
B37	176	242	176	138	206
B38	176	242	176	138	206
B41	176	242	176	138	206
B42	176	242	158	138	209
B46	176	242	176	138	206
B58	176	242	176	138	206
B59	176	242	176	138	206
B60	176	242	176	138	206
B61	176	242	179	138	206
B62	176	242	176	138	206
B63	176	242	176	138	206
B64	176	242	179	138	206
B65	176	242	176	138	206
B66	176	242	176	138	206
B67	176	242	176	138	206
B68	176	242	158	138	209
B69	176	242	176	138	206
B70	176	242	176	138	206
B71	176	242	176	138	206
B72	176	242	158	138	209
B74	176	242	158	138	209
B75	176	242	176	138	206
B76	176	242	179	138	206
B77	176	242	179	138	206
B79	176	242	176	138	206
B80	176	242	176	138	206
B82	176	242	158	138	206
B83	176	242	176	138	206
B84	176	242	176	138	206
B85	176	242	176	138	206
B86	176	242	176	138	206
B87	176	242	176	138	206
B89	176	242	176	138	206
B90	176	242	176	138	206
B91	176	242	158	138	209
B92	176	242	179	138	206
B94	176	242	158	138	209
B95	176	242	158	138	209
B97	176	242	158	138	209
B99	176	242	158	138	209
B100	176	242	176	138	206
B101	176	242	176	138	209
B103	176	242	158	138	209
B105	176	242	176	138	206
B106	176	242	176	138	206
B107	176	242	176	138	206
B108	176	242	176	138	206
B109	176	242	158	138	209

B110	176	242	158	138	209
B111	176	242	158	138	209
B114	176	242	176	138	206
B115	176	242	176	138	206
B116	176	242	158	138	206
B118	176	242	176	138	206
B119-a	176	242	176	138	206
B119-b	176	242	176	138	206
B120-b	176	242	176	138	206
B121-a	176	242	176	138	206
B121-b	176	242	176	138	206
B122-a	176	242	176	138	206
B122-b	176	242	176	138	206
B123-a	176	242	176	138	206
B124-a	176	242	176	138	206
B124-b	176	242	176	138	206
B125-a	176	242	158	138	209
B125-b	176	242	158	138	209
B126-a	176	242	158	138	206
B126-b	176	242	158	138	206
B127-a	176	242	176	138	206
B127-b	176	242	176	138	206
B128-a	176	242	176	138	206
B128-b	176	242	176	138	206
B129	176	242	158	138	209
B130-a	176	242	176	138	206
B130-b	176	242	176	138	206
B131	176	242	158	138	206
B132-a	176	242	158	138	206
B132-b	176	242	158	138	206
B133	176	242	176	138	206
B135	176	242	158	138	206
B136-a	176	242	158	138	206
B136-b	176	242	158	138	206
B137	176	242	176	138	209
B138-a	176	242	176	138	209
B138-b	176	242	176	138	209
B140-a	176	242	176	138	209
B140-b	176	242	176	138	209
B141-a	176	242	176	138	209
B141-b	176	242	176	138	209
B141-c	176	242	176	138	209
B141-d	176	242	176	138	209
B141-e	176	242	176	138	209
B143	176	242	176	138	209
B144-a	176	242	176	138	209
B144-b	176	242	176	138	209
B146	176	242	176	138	209
B147	176	242	176	138	209
B148	176	242	176	138	209

B149-a	176	242	176	138	209
B149-b	176	242	176	138	209
B150-a	176	242	176	138	206
B150-b	176	242	176	138	206
B150-c	176	242	176	138	206
B151-a	176	242	176	138	209
B151-b	176	242	176	138	209
B151-c	176	242	176	138	209
B152-a	176	242	176	138	209
B152-b	176	242	176	138	209
B153-a	176	242	158	138	206
B153-b	176	242	158	138	206
B154	176	242	158	138	209
B155	176	242	176	138	206
B156-b	176	242	176	138	206
B157-a	176	242	176	138	206
B157-b	176	242	158	138	209
B158-a	176	242	176	138	206
B158-b	176	242	176	138	206
B158-c	176	242	176	138	206
B158-d	176	242	176	138	206
B159-a	176	242	176	138	206
B159-b	176	242	158	138	206
B160-b	176	242	176	138	206
B161	176	242	158	138	206
B162-b	176	242	158	138	209
B163-a	176	242	158	138	209
B163-b	176	242	158	138	209
B164-a	176	242	176	138	206
B164-b	176	242	176	138	206
B165	176	242	176	138	206
B167-a	176	242	176	138	206
B167-b	176	242	158	138	209
B168	176	242	176	138	206
B169-b	176	242	176	138	206
B170-a	176	242	176	138	206
B171-b	176	242	158	138	209
B172-a	176	242	158	138	206
B175	176	242	176	138	206
B176	176	242	176	138	206
B177-b	176	242	176	138	206
B179-a	176	242	158	138	209
B179-b	176	242	158	138	209
B180-b	176	242	158	138	209
B181-b	176	242	158	138	209
B182	176	242	158	138	206
B183	176	242	176	138	206
B184-b	176	242	158	138	206
B185	176	242	158	138	206
B186-a	176	242	176	138	209

B186-b	176	242	176	138	209
B187-a	176	242	176	138	203
B187-b	176	242	158	138	206
B188	176	242	176	135	206
B189	176	242	176	135	206
B190	176	242	158	138	206
B191-a	176	242	176	138	206
B191-b	176	242	176	138	206
B192	176	242	176	138	206
B193	176	242	176	138	206
B194	176	242	176	138	206
B195	176	242	176	138	206
B196	176	242	176	138	206
B197	176	242	158	138	209
B198	176	242	176	138	206
B199	176	242	176	138	206
B200	176	242	176	138	206
B201	176	242	158	138	209
B202	176	242	176	138	206
B203	176	242	158	138	206
B204	176	242	176	138	206
B205	176	242	176	138	206
B206	176	242	158	138	206
B207-a	176	242	179	138	206
B207-b	176	242	179	138	206
B209-a	176	242	179	138	206
B209-b	176	242	179	138	206
B210	176	242	179	138	206
B211	176	242	176	138	206
B212-a	176	242	176	138	206
B212-b	176	242	176	138	206
B213	176	242	179	138	206
B214-a	176	242	179	138	206
B214-b	176	242	179	138	206
B215	176	242	179	138	206
B216	176	242	176	138	206
B217-a	176	242	176	138	206
B217-b	176	242	179	138	206
B218-a	176	242	179	138	206
B218-b	176	242	179	138	206
B219-a	176	242	179	138	206
B219-b	176	242	179	138	206
B219-c	176	242	179	138	206
B220-a	176	242	179	138	206
B220-b	176	242	176	138	206
B221	176	242	179	138	206
B222-a	176	242	176	138	206
B222-b	176	242	176	138	206
B223-a	176	242	176	138	206
B223-b	176	242	176	138	206

B224-a	176	242	179	138	206
B225-a	176	242	179	138	206
B225-b	176	242	179	138	206
B226-a	176	242	179	138	206
B226-b	176	242	179	138	206
B227-a	176	242	176	138	206
B227-b	176	242	179	138	206
B228	176	242	176	138	206
B229-a	176	242	179	138	206
B229-b	176	242	179	138	206
B230-a	176	242	179	138	206
B230-b	176	242	179	138	206
B231	176	242	179	138	206
B234	176	242	179	138	206
B235	176	242	179	138	206
B236-a	176	242	176	138	206
B236-b	176	242	176	138	206
B237-a	176	242	179	138	206
B237-b	176	242	179	138	206
B239-a	176	242	179	138	206
B239-b	176	242	179	138	206
B240-a	176	242	176	138	206
B240-b	176	242	176	138	206
B242	176	242	176	138	206
B244-a	176	242	176	138	206
B244-b	176	242	176	138	206
B245	176	242	176	138	209
B246	176	242	176	138	206

Powdery mildew-infected apple leaf samples with *Ampelomyces*

b13	176	254	176	138	206
b20	176	254	176	138	206
b25	176	254	158	138	206
b26	176	254	158	138	206
b27	176	242	176	138	206
b30	176	242	176	138	206
b31	176	242	176	138	206
b33	176	242	158	138	206
b34	176	242	176	138	206
b35	176	242	176	138	206
b36	176	242	176	138	206
b41	176	254	158	138	206
b44	176	254	176	138	206
b46	176	254	176	138	206
b49	176	254	158	138	206
b51	176	242	176	138	206
b52	176	254	176	138	206
b55	176	254	176	138	206
b58	176	254	176	138	206
b59	176	254	176	138	206

b60	176	254	176	138	206
b62	176	242	176	138	206
b65	176	254	176	138	206
b66	176	254	176	138	206
b69	176	254	176	138	206
b74	176	254	176	138	206
b76	176	254	176	138	206
b79	176	254	176	138	206
b81	176	254	176	138	206
b85	176	254	176	138	206
b87	176	254	176	138	206
b88	176	254	176	138	206
b92	176	254	158	138	209
b94	176	254	158	138	209
b96	176	254	176	138	206
b98	176	254	176	138	206
b99	176	254	176	138	206
b100	176	254	176	138	206
b102	176	254	176	138	206
b107	176	254	176	138	206
b110	176	254	176	138	206
b111	176	254	173	138	206
b113	176	254	176	138	206
b117	176	254	176	138	206
b119	176	254	173	138	206
b121	176	254	176	138	206
b122	176	254	176	138	206
b123	176	254	176	138	206
b124	176	254	176	138	206
b125	176	254	176	138	206
b128	176	257	176	138	206
b129	176	254	176	138	206
b130	176	254	176	138	206
b131	176	254	176	138	206
b132	176	254	176	138	206
b133	176	254	176	138	206
b134	176	254	176	138	206
b137	176	254	176	138	206
b139	176	254	176	138	206
b140	176	254	176	138	206
b143	176	254	158	138	209
b144	176	254	176	138	206
b146	176	242	158	138	206
b147	176	254	158	138	209
b153	176	254	176	138	206
b154	176	254	176	138	206
b155	176	254	158	138	209
b157	176	254	158	138	206
b164	176	254	176	138	206
b166	176	254	158	138	206

b167	176	254	176	138	206
b169	176	254	179	138	206
b170	176	254	176	138	206
b171	176	254	179	138	206
b174	176	254	179	138	206
b175	176	254	176	138	206
b177	176	254	179	138	206
b178	176	254	176	138	206
b182	176	254	176	138	206
b183	176	254	176	138	206
b185	176	254	179	138	206
b186	176	254	176	138	206
b190	176	254	179	138	206
b192	176	254	176	138	206
b193	176	254	176	138	206
b195	176	254	176	138	206
b196	176	254	176	138	206
b197	176	254	176	138	206
b199	176	254	176	138	206
b201	176	254	176	138	206
b205	176	254	176	138	206
b206	176	254	176	138	206
b207	176	254	176	138	206
b209	176	254	176	138	206
b210	176	254	176	138	206
b211	176	254	176	138	206
b212	176	254	179	138	206
b217	176	254	179	138	206
b218	176	254	179	138	206
b219	176	254	179	138	206
b220	176	254	176	138	206
b222	176	254	179	138	206
b223	176	254	179	138	206
b225	176	254	179	138	206
b226	176	254	179	138	206
b227	176	254	179	138	206
b228	176	254	179	138	206
b229	176	254	179	138	206
b230	176	254	179	138	206
b231	176	254	179	138	206
b232	176	254	179	138	206
b233	176	254	176	138	206
b234	176	254	176	138	206
b235	176	254	176	138	206
b236	176	254	176	138	206
b237	176	254	176	138	206
b238	176	254	176	138	206
b241	176	242	176	138	206
b242	176	266	176	138	206
b243	176	242	176	138	206

b244	176	242	176	138	206
b245	176	242	176	138	206
b246	176	242	176	138	206
b248	176	263	176	138	206
b249	176	254	176	138	206
b250	176	254	176	138	206
b251	176	254	176	138	206
b252	176	254	176	138	206
b253	176	254	176	138	206
b256	176	254	176	138	206
b257	176	254	176	138	206
b258	176	254	176	138	206
b261	176	242	176	138	206
b263	176	263	176	138	206
b266	176	242	176	138	206
b267	176	242	176	138	206
b268	176	242	176	138	206
b269	176	242	176	138	206
b271	176	242	176	138	206
b273	176	254	176	138	206
b275	176	242	176	138	206
b282-a	176	254	176	138	206
b285-a	176	254	158	138	206
b287-a	176	254	176	138	206
b288-c	176	254	176	138	206
b291	176	254	176	138	206
b297	176	242	176	138	209
b299	176	254	176	138	206
b301	176	242	176	138	206
b304	176	242	176	138	206
b307	176	254	176	138	206
b310	176	242	158	138	206
b315	176	254	158	138	206
b318	176	242	158	138	206
b324	176	242	158	138	206
b326	176	254	158	138	206
b331	176	242	158	138	206
b333	176	254	158	138	206
b339	176	254	158	138	206
b342	176	254	158	138	206
b344	176	254	158	138	206
b346	176	242	158	138	206
**Ampelomyces strains isolated from other powdery mildew species**					
BV2	194	254	173	132	239
BV4-b	194	254	179	132	239
BV4-c	194	254	179	132	239
RA1-b	194	269	179	132	239
RA2-a	194	296	179	132	230
RA2-d	194	269	179	132	230

MA1-c	194	254	179	132	239
MA5	194	257	179	132	239
MA6-b	194	254	179	132	239
MA7-a	194	257	179	132	239
MA7-b	194	257	179	132	239
MA8	194	254	179	132	239
MA-H	194	254	179	132	239
RS1-a	194	239	179	132	239
RS2-a	194	239	179	132	239
RS2-b	194	239	179	132	239
RS3-a	194	239	179	132	239
RS3-b	194	239	179	132	239
PN1-a	194	266	179	132	239
PN2	194	272	179	132	239
PN3	194	272	179	132	236
PN4-a	194	272	179	132	236
PN4-b	194	272	179	132	236
XL1-a	203	263	176	132	221
XL1-b	203	263	176	132	221
XL2-b	212	257	179	132	221
XL3-a	212	257	179	132	221
XL3-b	212	257	179	132	221
XL4-a	212	257	179	132	221
XL4-b	179	257	179	132	221
XL4-c	212	257	179	132	221
LV2-b	194	242	176	132	236
TP1	194	239	176	132	230
TP3	194	272	176	132	239
TP4	194	290	179	132	239
TP5	194	242	179	132	239
TR1	194	272	179	132	239
H14	194	239	176	132	230
GS1	194	242	176	132	239
GY-a	194	278	179	132	203
GY-b	194	278	179	132	203
GY-c	194	278	179	132	203
GL	194	242	179	132	239
ALA1-a	194	257	176	132	251
Aq SA	194	269	170	132	245
HMLAC226	194	239	176	132	221
G2	194	281	179	132	230
DSM2222	194	239	176	132	230
AQ10	194	245	182	132	242
GYER	176	206	179	126	224
GW	176	242	179	138	206
***Powdery mildew-infected leaf samples, other than apple leaves, containing Ampelomyces* pycnidia**					
tp2	194	239	179	132	239
tp13	194	239	176	132	230
tp20	194	272	179	132	230

tp21	194	242	179	132	239
tp24	194	272	176	132	239
tp25	194	302	179	132	239
