# Supplementary material for: Host Phenology and Geography as Drivers of Differentiation in Generalist Fungal Mycoparasites
Source: PLoS One. 2015 Mar 24;10(3):e0120703. doi: 10.1371/journal.pone.0120703 (PMC4372539; doi:10.1371/journal.pone.0120703)
Supplement: S3 Table — (PDF) [file pone.0120703.s007.pdf]

Table S3: genotypes of *Ampelomyces* strains isolated from different mycohosts in autumn, genotyped with eight microsatellite markers

	Lk3c	Lk7d	Lk7c	Lk3a	Lk10c	Lk3b	Lk7f	Lk10d
***Ampelomyces* strains isolated from grass powdery mildew (*Blumeria graminis*)**								
BgrA	155	152	194	269	179	132	195	239
BgrB	155	152	194	269	179	132	195	239
BgrC	155	152	194	269	179	132	195	239
BgrD	155	152	194	269	179	132	195	239
BgrE	155	152	194	269	179	132	195	239
BgrF	155	152	194	269	179	132	195	239
BgrG	155	152	194	269	179	132	195	239
BgrH	155	152	194	269	179	132	195	239
BgrI	104	170	194	296	149	132	209	200
KACC 43563	155	152	176	269	179	132	195	239
KACC 44850	104	158	176	296	182	132	195	203
KACC 44851	155	152	194	269	179	132	195	239
***Ampelomyces* strains isolated from grapevine powdery mildew (*Erysiphe necator*)**								
Vitis42	107	152	194	242	176	132	187	239
Vitis55	107	152	194	242	179	132	189	251
Vitis56	116	152	194	242	179	132	189	230
Vitis60	107	152	194	254	179	132	195	239
Vitis72	158	152	194	272	173	132	187	248
Vitis76	113	146	194	242	179	132	189	239
Vitis79	134	152	194	242	179	132	189	239
Vitis81	161	152	194	254	179	132	189	239
Vitis98	107	176	212	242	176	132	189	224
SF423 (ATCC200[illegible]	107	176	176	236	176	132	189	188
Ampelomyces* strains isolated from *Arthrocladiella mougeotii* infecting *Lycium halimfolium								
A1 (ATCC201056)	107	176	212	242	179	132	249	212
A8	122	176	203	263	176	132	187	221
A10-a	122	176	212	263	179	132	185	221
A10-c	122	176	212	263	179	132	185	221
A11-a	122	176	203	263	176	132	187	221
A11-b	122	176	203	263	176	132	187	221
A11-c	122	176	203	263	176	132	187	221
A12-a	122	176	203	263	176	132	187	221
A12-c	122	176	203	263	176	132	187	221
A13-a	122	176	203	263	176	132	187	221
A13-b	122	176	203	263	176	132	187	221
A13-c	122	176	203	263	176	132	187	221
A14-a	122	176	203	263	176	132	187	221
A14-b	122	176	203	263	176	132	187	221
A14-c	122	176	203	263	176	132	187	221
A14-d	122	176	203	263	176	132	187	221
A15-a	161	176	212	257	179	132	201	221
A17	161	176	212	257	179	132	201	221
A18-a	131	188	203	242	176	132	253	221
A19-a	107	188	212	242	176	132	205	212

A19-b	107	188	212	242	176	132	205	212
A19-c	107	188	212	242	176	132	205	212
A20-a	107	188	212	242	176	132	201	212
A20-b	107	188	212	242	176	132	201	212
A20-c	107	188	212	242	176	132	201	212
A26	119	152	212	263	179	132	189	212
A33-c	131	188	203	263	179	132	191	239
A33-e	131	188	203	263	179	132	191	239
A34-d	131	188	203	263	179	132	191	239
A38-c	107	176	188	209	197	126	187	221
A39-a	128	176	194	257	176	132	189	212
A39-c	131	176	212	263	176	132	221	233
A41-b	128	176	194	257	176	132	189	212
A41-c	134	176	203	257	176	132	223	212
A52-b	134	188	194	248	179	132	187	224
A53-a	104	188	194	212	149	132	219	209
A53-b	104	188	194	212	149	132	199	209
A53-c	104	188	194	212	149	132	199	209
A54-a	104	188	194	212	149	132	199	209
A54-b	104	188	194	212	149	132	199	209
A54-c	104	188	194	212	149	132	219	209
A55-a	104	188	194	212	149	132	199	209
A55-b	104	188	194	212	149	132	199	209
A55-c	104	188	194	212	149	132	219	209
A57-a	113	152	194	242	179	132	189	239
A57-b	113	152	194	242	179	132	189	239
A60	107	152	194	269	179	132	185	239
A61-a	158	152	194	269	179	132	185	239
A61-b	158	152	194	269	179	132	185	239
A61-c	158	152	194	269	179	132	185	239
A62-a	158	152	194	269	179	132	185	239
A62-b	158	152	194	269	179	132	185	239
A62-c	158	152	194	269	179	132	185	239
A64-a	158	152	194	269	179	132	185	239
A64-b	158	152	194	269	179	132	185	239
A64-c	158	152	194	269	179	132	185	239
A69-a	107	176	212	263	179	132	189	212
A69-b	107	176	212	263	179	132	189	212
A70-a	107	176	212	263	179	132	189	212
A70-b	107	176	212	263	179	132	189	212
A71-a	107	176	212	263	179	132	189	212
A71-b	107	176	212	263	179	132	189	212
A72-a	107	176	212	263	179	132	189	212
A72-b	107	176	212	263	179	132	189	212
A72-c	107	176	212	263	179	132	189	212
A72-d	107	176	212	263	179	132	189	212
A75-a	107	176	212	263	179	132	189	212
A75-c	107	176	212	263	179	132	189	212
A76-a	107	176	212	263	179	132	189	212
A76-b	107	176	212	263	179	132	189	212

A76-c	107	176	212	263	179	132	189	212
A77-a	107	176	212	263	179	132	189	212
A77-b	107	176	212	263	179	132	189	212
A81-a	128	176	203	254	176	132	189	224
A82-a	134	188	212	242	179	132	189	224
A82-b	134	188	212	242	179	132	189	224
A83	134	176	212	257	179	132	223	224
A84-b	152	176	212	248	176	132	249	221
A84-c	152	176	212	248	176	132	249	224
A85	134	188	203	242	176	132	189	224
A86-a	134	176	212	257	179	132	225	224
A86-c	134	176	194	257	176	132	185	224
A86-d	134	176	212	242	176	132	221	212
A86-e	131	176	212	242	173	132	251	224
A87-a	131	176	221	242	179	132	189	230
A87-b	131	176	221	242	179	132	189	230
A87-c	134	176	194	257	176	132	185	224
A87-d	131	176	221	242	179	132	189	230
A88-a	137	176	212	242	179	132	253	224
A88-b	134	188	212	257	179	132	189	224
A88-c	134	176	212	242	176	132	221	212
A88-d	137	176	212	242	179	132	253	224
A89-a	131	176	212	242	173	132	251	224
A89-b	131	176	212	242	173	132	251	224
A89-c	134	188	212	257	179	132	189	224
A89-d	131	176	212	242	173	132	251	224
A90-c	107	176	212	242	176	132	221	212
A90-d	134	176	212	242	176	132	221	212
A91-a	131	176	212	242	173	132	251	224
A91-b	131	176	212	242	173	132	251	224
A91-c	134	176	194	257	176	132	185	224
A91-d	131	176	212	242	173	132	251	224
A92-a	140	152	194	254	179	132	191	242
A92-b	140	152	194	254	179	132	191	242
A92-c	140	152	194	254	179	132	191	242
A92-d	140	152	194	254	179	132	191	242
A92-e	140	152	194	254	179	132	191	242
A94-a	119	152	194	272	179	132	189	239
A96-a	119	152	194	272	179	132	191	239
A96-b	119	152	194	272	179	132	191	239
A96-c	119	152	194	272	179	132	191	239
A96-d	119	152	194	272	179	132	191	239
A99	107	152	188	206	176	126	189	221
A101	131	158	194	242	176	132	189	212
A102-a	134	176	212	257	176	132	189	224
A102-b	134	176	212	257	176	132	189	224
A102-c	134	176	212	257	176	132	189	224
A103	119	152	194	272	179	132	189	239
A104-a	101	188	185	209	149	132	199	215
A104-b	134	176	212	257	176	132	189	224

A104-c	134	176	212	257	176	132	189	224
A105	101	188	185	209	149	132	199	215
A108-a	107	176	212	254	176	132	189	212
A108-b	107	176	212	254	176	132	189	212
A108-d	107	176	212	254	176	132	189	212
A110-b	107	152	188	206	176	126	189	221
A110-c	146	176	212	269	179	132	249	224
A110-d	146	176	212	269	179	132	249	224
A111-a	131	176	212	269	179	132	247	224
A111-b	107	152	188	206	179	126	247	221
A113-a	146	176	212	269	179	132	249	224
A113-b	146	176	212	269	179	132	249	224
A115	107	176	212	254	176	132	189	212
A120	161	152	194	269	179	132	185	239

***Ampelomyces* strains isolated from other powdery mildew species**

BV2	161	152	194	254	173	132	189	239
BV4-b	119	152	194	254	179	132	185	239
BV4-c	119	152	194	254	179	132	185	239
RA1-b	137	146	194	269	179	132	193	239
RA2-a	113	146	194	296	179	132	191	230
RA2-d	131	152	194	269	179	132	191	230
MA5	134	146	194	257	179	132	189	239
MA6-b	134	146	194	254	179	132	187	239
MA7-a	134	146	194	257	179	132	189	239
MA7-b	134	146	194	257	179	132	189	239
MA-H	134	146	194	254	179	132	187	239
RS1-a	128	152	194	239	179	132	189	239
RS2-a	128	152	194	239	179	132	189	239
RS2-b	128	152	194	239	179	132	189	239
RS3-a	128	152	194	239	179	132	189	239
RS3-b	128	152	194	239	179	132	189	239
PN1-a	143	152	194	266	179	132	187	239
PN2	125	152	194	272	179	132	187	239
PN3	125	152	194	272	179	132	189	236
PN4-a	125	152	194	272	179	132	189	236
PN4-b	125	152	194	272	179	132	189	236
XL1-a	122	176	203	263	176	132	187	221
XL1-b	122	176	203	263	176	132	187	221
XL2-b	161	176	212	257	179	132	201	221
XL3-a	161	176	212	257	179	132	201	221
XL3-b	161	176	212	257	179	132	201	221
XL4-a	161	176	212	257	179	132	201	221
XL4-b	161	176	179	257	179	132	201	221
XL4-c	161	176	212	257	179	132	201	221
LV2-b	125	152	194	242	176	132	189	236
TP1	119	152	194	239	176	132	189	230
TP3	161	152	194	272	176	132	189	239
TP4	116	152	194	290	179	132	189	239
TP5	125	152	194	242	179	132	189	239

TR1	119	146	194	272	179	132	189	239
H14	119	152	194	239	176	132	189	230
GS1	119	152	194	242	176	132	189	239
GY-a	104	164	194	278	179	132	189	203
GY-b	104	164	194	278	179	132	189	203
GY-c	104	164	194	278	179	132	189	203
GL	116	146	194	242	179	132	193	239
ALA1-a	113	152	194	257	176	132	191	251
Aq SA	116	152	194	269	170	132	191	245
G2	113	152	194	281	179	132	189	230
HMLAC226	113	152	194	239	176	132	193	221
DSM 2222	119	152	194	239	176	132	189	230
AQ10	125	158	194	245	182	132	193	242
**Powdery mildew-infected leaf samples, other than apple leaves, containing *Ampelomyces* pycnid**								
tp2	119	152	194	239	179	132	189	239
tp13	119	152	194	239	176	132	189	230
tp20	116	152	194	272	179	132	189	230
tp21	119	152	194	242	179	132	189	239
tp24	107	152	194	272	176	132	191	239
tp25	128	152	194	302	179	132	189	239

ia
